# Probing Guanidino Pendant or Bridged Groups in Cyclic Antimicrobial Peptides Derived from Temporin L: A Strategy to Improve Efficacy against Gram-Negative Bacteria

**DOI:** 10.1021/acs.jmedchem.5c01984

**Published:** 2025-12-01

**Authors:** Rosa Bellavita, Ida Boccino, Maria Rosa Loffredo, Sara Palladino, Floriana Cappiello, Carlo Vetrano, Eeva Tortellini, Vincenzo Mazzarella, Salvatore Di Maro, Stefania Galdiero, Bruno Casciaro, Paolo Grieco, Maria Luisa Mangoni, Francesco Merlino

**Affiliations:** † Department of Pharmacy, University of Naples Federico II, via Domenico Montesano 49, 80131 Naples, Italy; ‡ Department of Biochemical Sciences, Laboratory Affiliated to Istituto Pasteur Italia-Fondazione Cenci Bolognetti, Sapienza University of Rome, p.le Aldo Moro 5, Rome 00185, Italy; § DiSTABiF, University of Campania Luigi Vanvitelli, via Vivaldi 43, 81100 Caserta, Italy

## Abstract

The rise of antibiotic resistance underscores the urgent need for new antimicrobial agents. Antimicrobial peptides (AMPs), such as temporins, offer broad-spectrum activity through unique mechanisms but are often limited by cytotoxicity and poor stability. To improve the Gram-negative activity-to-toxicity ratio, we designed a focused library of cyclic Temporin L (TL) analogues bearing an additional positively charged guanidino group, introduced either as a side-chain pendant or as a bridged functionality. While guanidino modifications have been studied in side-chain peptide contexts, this represents the first application of guanidino-based bridging in AMP design. Among the synthesized compounds, four (*e*.*g*., **2**, **6**, **7** and **12**) were selected based on their combined antimicrobial potency and low cytotoxicity toward human keratinocytes, emerging as the most structurally representative candidates of the introduced modifications. Further characterization provided an integrated view of their biological properties, highlighting guanidino-based cyclic temporins as attractive agents and a framework for developing next-generation therapeutics against resistant and biofilm-associated infections.

## Graphical abstract

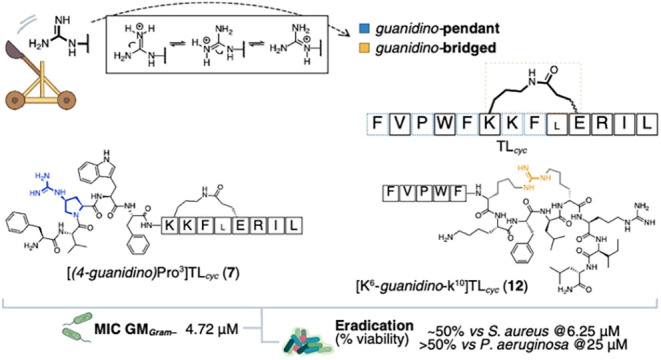

## Introduction

In the ongoing quest for effective antimicrobial agents, naturally occurring peptides have emerged as promising therapeutic candidates, owing to their broad-spectrum activity, membrane-disruptive mechanisms, and remarkably low propensity for inducing resistance mechanisms in target pathogens.^1–3^ Among the diverse array of antimicrobial peptides (AMPs), Temporin L (TL), a 13-mer linear peptide (sequence: FVQWFSKFLGRIL) belonging to the temporins family, was originally isolated from the skin secretions of the European red frog *Rana temporaria* and garnered considerable attention for its potent and multifaceted antimicrobial profile.^4,5^ TL has been shown to exhibit activity against a broad spectrum of pathogens, including Gram-positive and Gram-negative bacteria, fungi, and even some viruses.^4,6–8^ Despite its high efficacy, TL’s clinical potential is limited by its cytotoxicity at microbicidal concentrations, which poses challenges for therapeutic applications as such.^5^

Structurally, TL adopts a random coil conformation in aqueous solution, but transitions to an α-helical structure in membrane-mimetic environments such as negatively charged sodium dodecyl sulfate (SDS) and zwitterionic dodecylphosphorylcholine (DPC) micelles, which mimic bacterial and mammalian membranes, respectively.^9,10^ NMR analyses in SDS micelles indicate a well-defined α-helix spanning residues 3 to 11, whereas the *N-* and *C-*termini appear more disordered (Figure 1, panel A).^11^ Conversely, DPC micelles induce a more extended α-helical conformation involving all residues. Moreover, structural studies in lipopolysaccharide (LPS) micelles, which mimic Gram-negative outer membranes, revealed that TL assumes an amphipathic helix, with hydrophobic/aromatic (W^4^, F^5^, L^9^, F^8^ and I^12^) and hydrophilic (Q^3^, S^6^, K^7^ and R^11^) residues segregated on opposite faces of the helix (Figure 1, panel B).^12^ TL is known to exert a membranolytic mechanism of action, a hallmark of many AMPs, and to inhibit biofilm formation and disrupt preformed biofilms of *Pseudomonas fluorescens*.^13^ Interestingly, recent studies have suggested an intracellular mechanism of action as well, specifically targeting the FtsZ protein in *Escherichia coli*, a key regulator of bacterial cell division.^14,15^ As a result of these properties, TL now has served as a template for developing novel analogues with improved therapeutic indices. To this end, various sequence modifications targeting its primary and/or secondary structure have been explored to reduce cytotoxicity while retaining or enhancing antimicrobial efficacy (Figure 1, panels C and D). For instance, [Pro^3^,dLeu^9^]­TL, generated by replacing Gln^3^ with Pro and inverting the stereochemistry of Leu^9^, showed reduced hemolysis while maintaining antimicrobial activity, particularly against Gram-positive pathogens.^9,10,16,17^ A further optimized variant, [Pro^3^,dLeu^9^ ,dLys^10^]­TL, demonstrated superior efficacy across Gram-negative [minimal inhibitory concentrations (MICs) ranging from 6.25 to 12.5 μM] and Gram-positive (MICs ranging from 3.12 to 12.5 μM) strains, and significantly reduced cytotoxicity up to 25 μM compared to its precursor [Pro^3^,dLeu^9^]­TL.^18^ In another study, Verma et al. introduced a single noncanonical amino acid, *e*.*g*., β-Leu, in place of both Leu^9^ and Gly^10^, creating L9βl-TL, which showed enhanced efficacy against planktonic and biofilm methicillin-resistant *Staphylococcus aureus* (MRSA) strain (MIC 3.1 μM for L9βl-TL *vs* 6.25 μM for TL) and reduced hemolysis.^19^ Similarly, Kumari et al. developed the analogue eTL (SW,Q3K,F8K-TL), which displayed potent activity, especially against Gram-negative strains such as *E. coli* and *Klebsiella pneumoniae* (MICs of 1.9 μM and 3.8 μM, respectively), while also being noncytotoxic.^20^ Intriguingly, eTL lacked a defined secondary structure and acted *via* a nonmembranolytic mechanism, with endotoxin-neutralizing capabilities. However, due to its linear sequence of natural amino acids, eTL was susceptible to protease degradation and presented a short serum half-life. To overcome this limitation, more recently the same group employed hydrocarbon stapling techniques to stabilize the peptide structure and enhance serum resistance.^21^ Among the resulting analogues, eTL[5–9] stood out for its strong antibacterial activity against MRSA (MIC, 3.6 μM), along with noncytotoxicity and stability in human serum (MIC, 7.2 μM in 20% serum). The structural characteristics of TL, comprising a linear array of amino acids with amphipathic α-helical architecture, have also previously served us providing a logical framework for designing the first cyclic analogues.^22^ We focused on side-chain-to-side-chain tethering, particularly within the *C*-terminal region of [Pro^3^,dLeu^9^]­TL to confer a chemical stabilization of the α-helical structure. The lactam bridge between residues in position 6 and 10 (originally Ser and Gly), yielded the first cyclic TL-derived AMP, herein named as TL_*cyc*_ (**1**), which showed enhanced stability, antimicrobial and antibiofilm activities.^22^ However, its performance against Gram-negative bacteria remained limited (MICs ranging from 3.12 to 100 μM) with respect to the linear reference peptide [Pro^3^,dLeu^9^]­TL.

**Figure 1:**
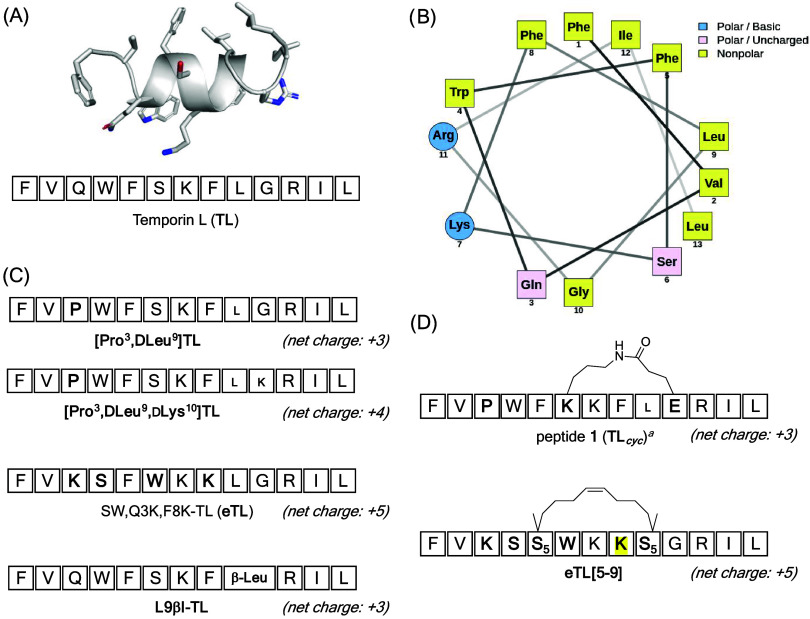
Structural and sequence modifications of TL and its analogs. (A) Native TL sequence and 3D NMR structure (PDB: 6GS5).^11^ (B) Helical wheel showing amphipathic residue distribution. (C) Linear analogs with substitutions and net charges indicated. (D) Cyclic analogs of TL, designed to enhance peptide stability and antimicrobial activity. Net charges were calculated by considering all peptides with a free *N*-terminal amine and a *C*-terminal amide. ^*a*^Peptide **1** (TL_cyc_) corresponds to peptide **12** in Bellavita et al.^22^

Based on these findings, this study examines targeted sequence alterations, such as inverting the stereochemistry of the cyclizing Glu^10^ residue and introducing additional positively charged moieties as guanidino groups in pendant or bridged positions. The guanidino group was selected based on its well-established role as a highly basic and chemically flexible motif, known to strengthen membrane interactions, increase antimicrobial potency, and improve target specificity in peptide-based structures.^23,24^ We reasoned that strategically introducing guanidino functionalities into the TL_cyc_ backbone could yield a new class of TL-derived antimicrobial peptides with enhanced electrostatic interactions, increased potency, and a broadened antimicrobial spectrum, particularly against Gram-negative pathogens. To test this hypothesis, we employed an integrated approach combining synthetic peptide chemistry, antibacterial assays, membrane interaction studies, and structural characterization, with the goal of refining the structure–activity relationship (SAR) guiding the function of these newly designed guanidino-based cyclic AMPs.

## Results and Discussion

### Design Strategy and Peptide Synthesis

The development of new TL_cyc_ analogues was inspired by previous modifications applied to linear TL-derived sequences, which had resulted in derivatives with enhanced activity against Gram-negative strains.^18,20^ Notably, such enhancements were often associated with an increased number of positive charges within the sequence. Based on SAR studies,^18,22^ we first focused on position 10, originally occupied by Gly in TL, as a previously reported replacement of Gly^10^ with d-Lys was critical in enhancing antimicrobial activity.^18^ Specifically, the resulting analogue [Pro^3^,dLeu^9^,dLys^10^]­TL demonstrated superior activity against *E. coli* and *Pseudomonas aeruginosa*, along with reduced cytotoxicity, compared to its l-Lys counterpart. In line with these findings, the cyclizing residue l-Glu at position 10 in TL_cyc_ was initially substituted with d-Glu, yielding peptide **2** (Table 1). Preliminary antimicrobial activity and cytotoxicity assays for peptide **2** showed improved activity against some Gram-negative strains such as *E. coli* and *P. aeruginosa*, while maintaining cytotoxicity comparable to reference peptide **1** ­(Tables 2 and 3). These results encouraged us to retain the stereoinversion at position 10 in subsequent derivatives. Based on the beneficial effects of d-Lys at position 10, a positive charge was also introduced albeit *via* a guanidino group. The guanidino group, known for its high p*K*_a_ values (p*K*_a1_ = 2.18, p*K*_a2_ = 9.09, p*K*_a3_ = 13.20 for arginine *vs* p*K*_a_ = 10.94 for lysine) due to efficient resonance stabilization of the charged protonated state, forms stable electrostatic interactions, including hydrogen bonds, salt bridges, cation-π interactions with negatively charged phosphodiester and phosphomonoester groups of membrane phospholipids.^25^

**Table 1:**
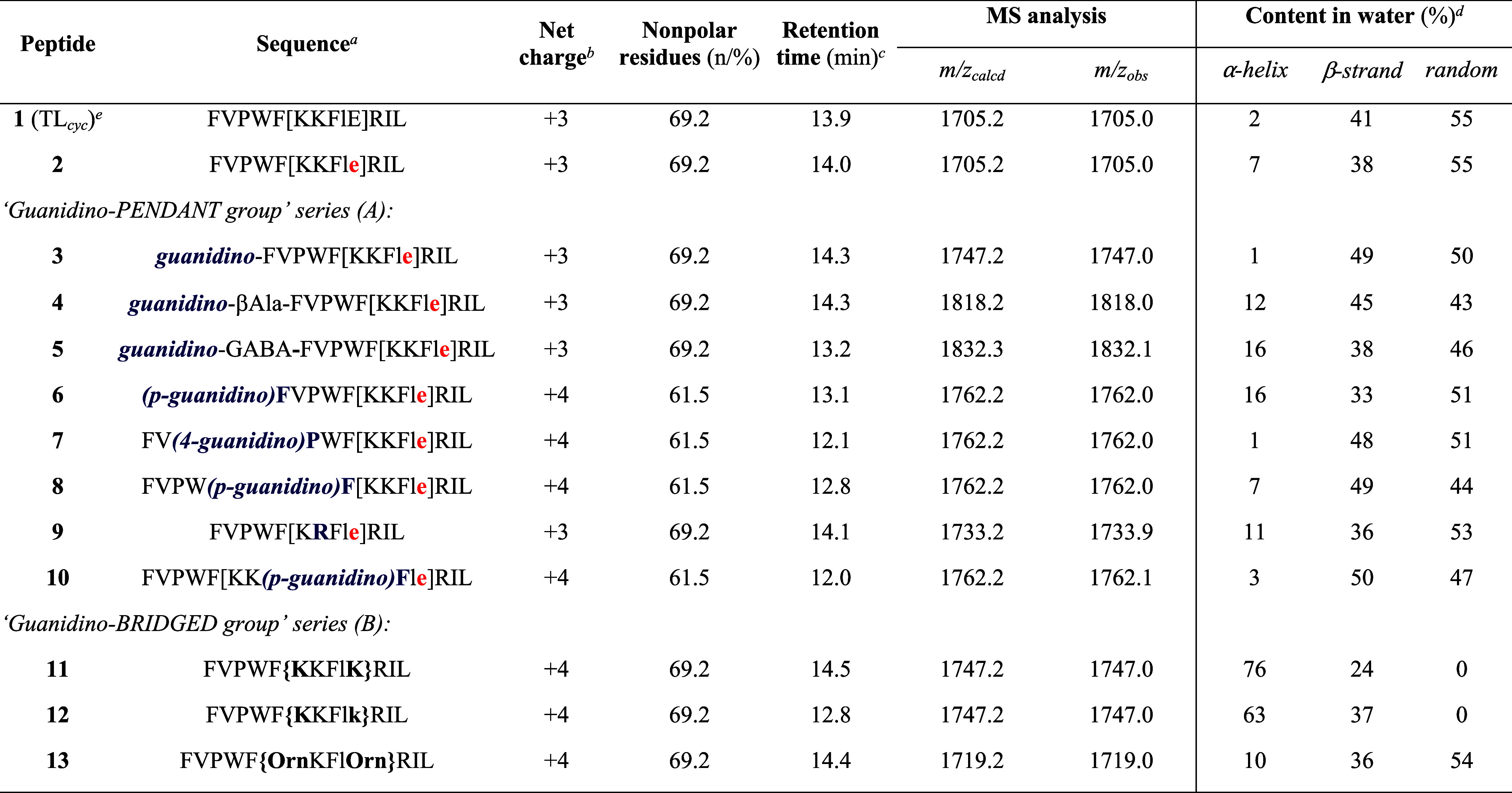
Sequences and Key Physicochemical Parameters of the Newly Designed Guanidino-Based TL_cyc_-Derived Peptides

a Designed modifications are highlighted: stereoinversion of Glu^10^ (red); guanidino-pendant group (blue); guanidino-bridged group ({}). Lowercase letters indicate the corresponding d-amino acids.

b Net charges were calculated as the sum of contributions from conventional positively charged residues (*e*.*g*., Lys, Arg), nonconventional amino acids bearing guanidino substituents, guanidino-bridged moieties, and/or the free *N*-terminal amino or guanidino group. The *C*-terminus was considered amidated, consistent with the synthesized structures.

c Retention times were determined by analytical HPLC, as described in the Experimental Section.

d Secondary structure content in H_2_O was predicted from CD spectra using BeStSel.

e Values reported for peptide **1** (TL_cyc_) correspond to those previously determined for peptide 12 reported in Bellavita et al.^22^

**Table 2:** Antimicrobial Activity of the Designed Guanidino-Based TL_cyc_-Derived Peptides

	antimicrobial activity (MIC in μM)										geometric mean^b^		
Cmpd	*S. aureus* ATCC 25923	*S. epidermidis* ATCC 12228	B. megaterium BM11	*S. agalactiae* ATCC 27591	*E. faecalis* ATCC 29212	*E. coli* ATCC 25922	*P. aeruginosa* ATCC 27853	*A. baumannii* ATCC 19606	*E. coli* D21	*P. aeruginosa* PAO1	GM_all_	GM_Gram+_	GM_Gram–_
**1** (TL_*cyc*_)	**3.12** ^a^	**3.12** ^a^	**1.56** ^a^	**3.12**	**6.25**	**25** ^a^	**100** ^a^	**3.12** ^a^	**12.5**	**25**	7.67	3.12	18.9
**2**	12.5	3.12	0.78	3.12	25	6.25	50	6.25	12.5	50	8.81	4.72	16.4
**3**	6.25	3.12	0.78	3.12	25	6.25	100	3.12	6.25	50	7.16	3.58	14.32
**4**	3.12	1.56	0.78	1.56	6.25	3.12	25	3.12	6.25	25	3.76	2.06	8.22
**5**	3.12	3.12	0.78	1.56	6.25	6.25	50	3.12	6.25	25	5.06	2.36	10.85
**6**	25	3.12	0.78	1.56	12.5	6.25	25	3.12	3.12	12.5	5.43	4.11	7.16
**7**	6.25	3.12	0.78	1.56	12.5	3.12	12.5	3.12	3.12	6.25	3.84	3.12	4.72
**8**	25	6.25	0.78	3.12	25	12.5	50	6.25	6.25	25	9.45	6.23	14.32
**9**	6.25	3.12	1.56	3.12	12.5	6.25	100	3.12	6.25	50	7.67	4.11	14.32
**10**	100	12.5	0.78	6.25	50	6.25	50	6.25	6.25	25	12.47	12.47	12.5
**11**	3.12	1.56	1.56	3.12	12.5	6.25	50	1.56	12.5	50	6.23	3.12	12.5
**12**	6.25	1.56	0.39	1.56	6.25	6.25	12.5	1.56	3.12	6.25	3.12	2.06	4.72
**13**	6.25	1.56	0.39	1.56	6.25	6.25	50	1.56	6.25	12.5	4.11	2.06	8.22
**Ciprofloxacin** (μg/mL)	0.5	0.25	0.12	0.5	0.5	0.01	0.5	1	0.01	0.12	*n*.*c*.	*n*.*c*.	*n*.*c*.

a MIC values previously determined for peptide 12 reported in Bellavita et al.^22^

b Geometric mean of the MICs of the peptides against all (GM_all_), Gram-positive (GM_Gram+_) and Gram-negative (GM_Gram–_) bacteria tested. n.c. = not calculated.

**Table 3:**
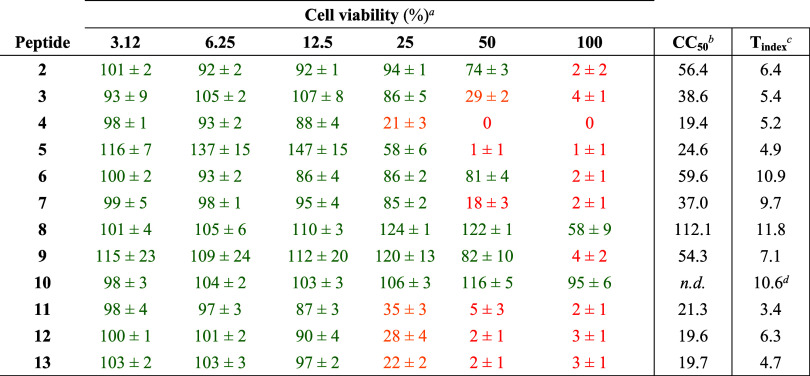
Cytotoxicity against Human Keratinocytes (HaCaT)

a Cell viability was assessed using the MTT assay after treatment with increasing concentrations of peptides (3.12, 6.25, 12.5, 25, 50, and 100 μM) after 24 h of exposure. Results are expressed as the percentage of viable cells relative to untreated controls and represent the mean ± standard error of the mean (SEM) of three independent experiments. Green, cell viability ≥ 50%; orange, cell viability from 49 to 20%; red, cell viability <20%.

b CC_50_ corresponds to the peptide concentration that cause 50% of cell death and was calculated using “Quest Graph LD50 Calculator”.

c *T*_index_ was calculated as the ratio of CC_50_/GM_all_.

d This value was calculated considering the cell viability after 2 h of exposure. n.d. = not determined.

In our design strategy, the guanidino group was incorporated either as pendant (“guanidino-pendant group” series A, peptides **3–10**) or bridged (“guanidino-bridged group” series B, peptides **11–13**) modification at synthetically accessible positions (Figure 2). For pendant modifications, we targeted residues such as Phe, Pro, and Lys, by using their commercially available guanylated counterparts, (*p*-guanidino)­Phe, (4-guanidino)­Pro and Arg, respectively. Additionally, we explored the *N*-terminal region to attach the guanidino group directly to the peptide backbone or separated by one or two methylene groups, such as *via* β-alanine (β-Ala) or *γ*-ammino butyric acid (GABA). For the bridged strategy, the guanidino group was integrated within the cyclizing motif, replacing the lactam bridge and thereby creating the first AMP analogues with such features. Indeed, to the best of our knowledge, guanidino-based bridge cyclic peptides between diaminoacyl side chains have been reported only in cyclic peptides with antinociceptive,^26^ anticancer,^27^ or low density lipoprotein receptor (LDLR)-binding activities,^28^ but not in antimicrobial peptide sequences. Finally, to optimize activity, we varied the cycle size using Lys or ornithine (Orn), combined with stereoinversion, aiming to identify the best-performing antimicrobial analogue. These modifications allowed us to evaluate the combined effects of stereoinversion and the introduction of a new positive charge on the antimicrobial efficacy, cytotoxicity and stability of these new derivatives.

**Figure 2:**
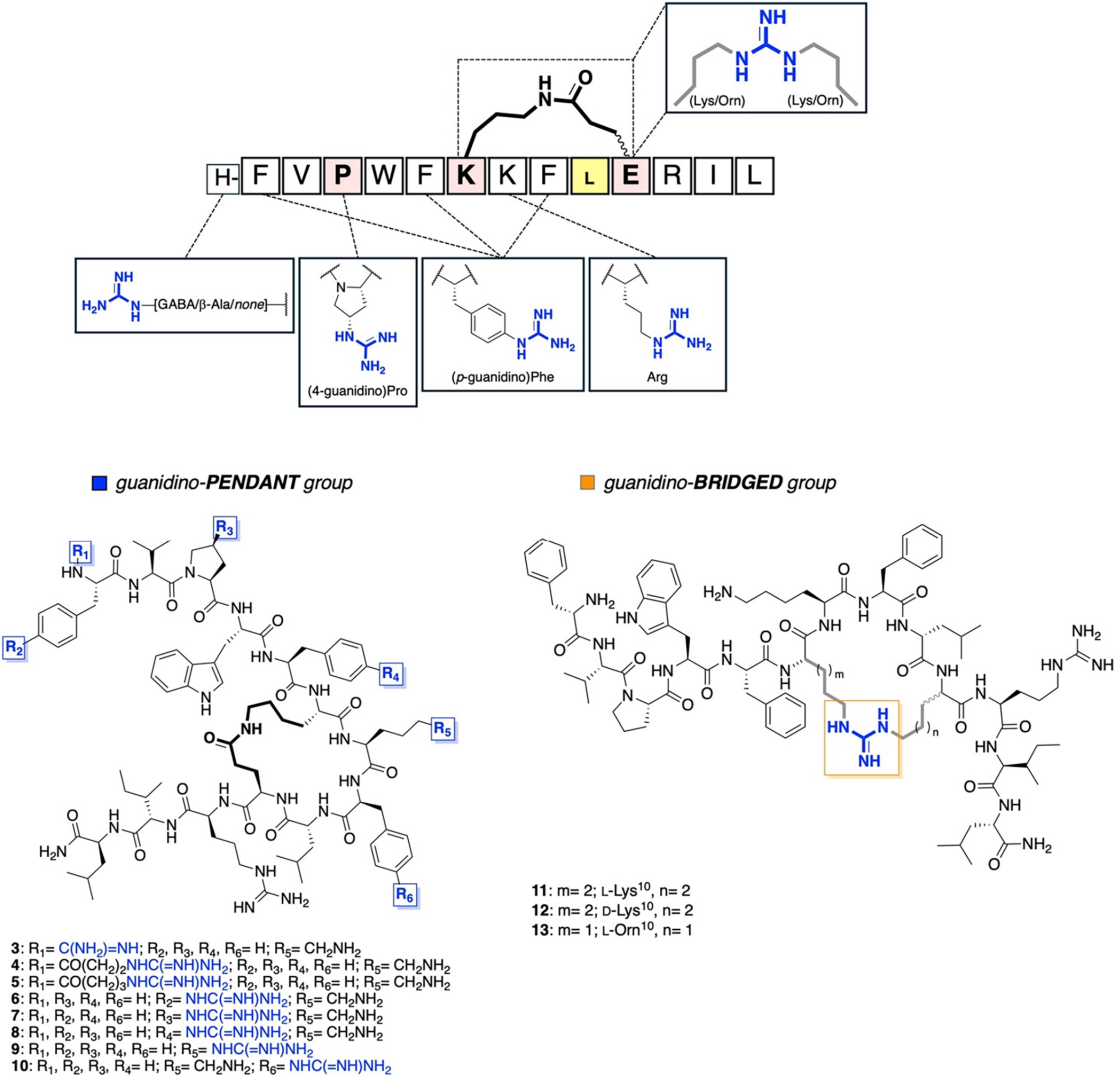
Design and structural representation of guanidino-modified analogs of TL_cyc_ (peptide **1**). Introduction of a guanidino group was done in key positions through guanidino-bearing side chains, such as (4-guanidino)­Pro, (*p*-guanidino)­Phe, and Arg, as well as guanidino-alkyl derivatives, introduced by GABA, β-Ala, or direct attachment. Chemical structures of guanidino-modified cyclic peptides presented in this study and divided into two series: (i) guanidino-pendant (blu) (peptides **2–10**) and (ii) guanidino-bridged (orange) (peptides **11–13**) groups.

As for the synthesis, linear peptides were assembled using the Fmoc-based ultrasonic-assisted solid-phase peptide synthesis (US-SPPS), as previously reported.^29–31^ Macrocyclization was then achieved through two distinct strategies, as illustrated in Scheme 1.^32,33^ For peptides **2–10**, a lactam bridge was introduced according to established procedures (Scheme 1, panel A).^22^ Peptide intermediates containing guanidine as pendant groups on side chain residues were first subjected to a palladium-catalyzed reaction to selectively remove allyl-based protecting groups from Lys^6^ and d-Glu^10^ (path 1). Lactam cyclization was subsequently carried out using (7-azabenzotriazol-1-yloxy)­trispyrrolidinophosphonium hexafluorophosphate (PyAOP) and 1-hydroxy-7-azabenzotriazole (HOAt), as coupling/additive reagents, in the presence of *N*,*N*-diisopropylethylamine (DIEA). Following cyclization, peptides **6–10** were directly cleaved from the resin. In contrast, peptides **3–5**, featuring a guanidino group at the *N*-terminus, were prior subjected to a guanylation reaction (path 2), as described elsewhere.^34^ This transformation was achieved using mercury­(II) chloride (HgCl_2_), 1,3-bis­(*tert*-butoxycarbonyl)-2-methyl-2-thiopseudourea, DIEA and *N*,*N*-dimethylformamide (DMF) under mild stirring conditions. To introduce a spacer between the peptide backbone and the guanidino group, β-Ala and GABA were incorporated in peptides **4** and **5**, respectively. The second macrocyclization strategy (Scheme 1, panel B) was applied to peptides **11–13**. This involved selective deprotection of the allyloxycarbonyl (Alloc) group from Lys/Orn^10^ side chains, followed by isothiocyanate formation using di­(2-pyridyl)­thionocarbonate (DPT) in dichloromethane (DCM) under gentle agitation.^26,32^ Next, the 4-methyltrityl (Mtt) protecting group was removed using a cocktail of DCM/TIS/TFA (94:5:1, v/v/v). The resulting free amine on the resin was then treated with triethylamine (TEA) in dry tetrahydrofuran (THF) to form thiourea. This was followed by the *S*-methylation reaction using a solution of iodomethane (CH_3_I) in DMF. Finally, nucleophilic substitution was performed using ammonium acetate (NH_4_OAc) in dimethyl sulfoxide (DMSO) with *N*-methylmorpholine (NMM) at 80 °C for 12 h.

**Scheme 1:**
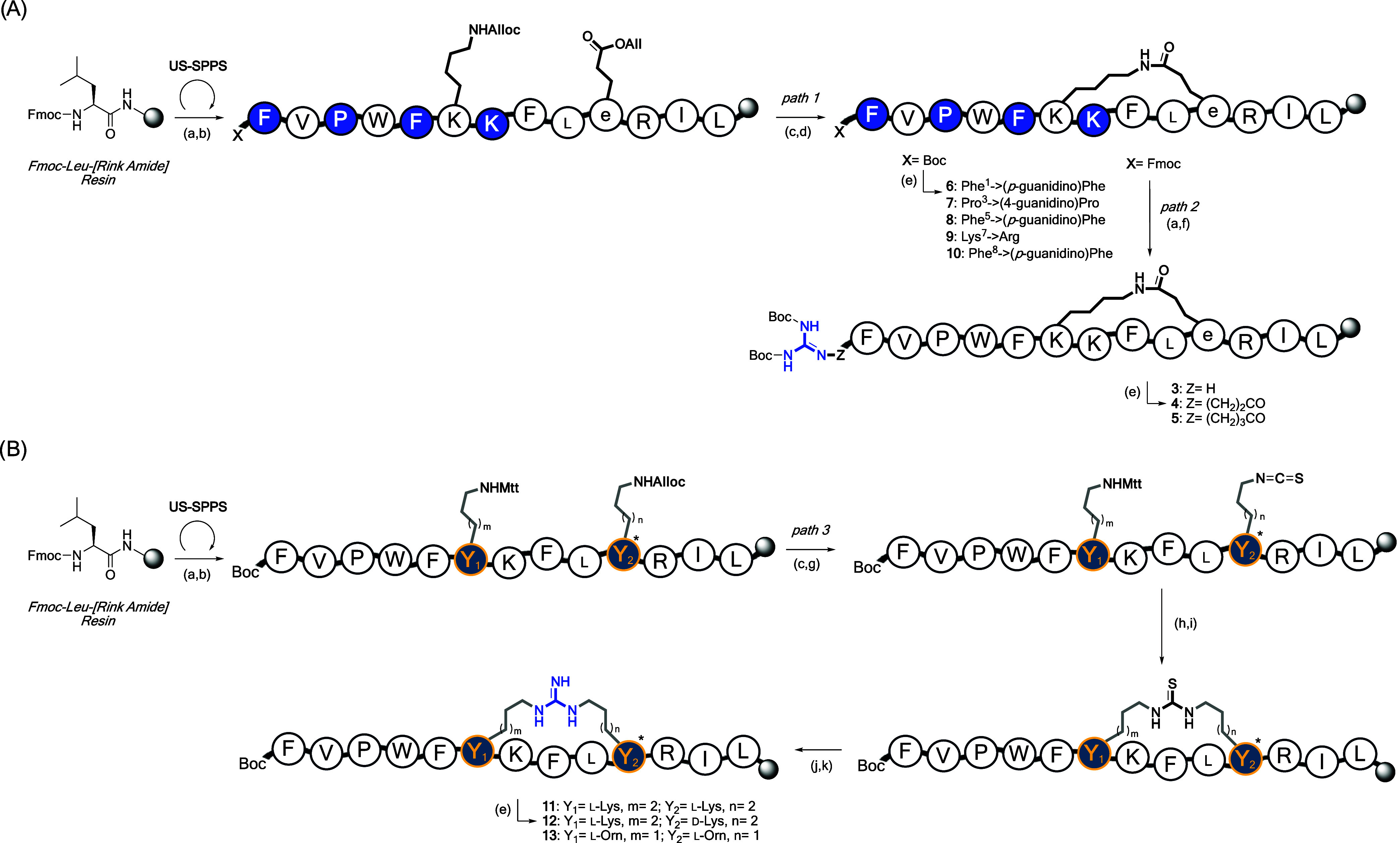
Synthetic Routes to Achieve Guanidino-Functionalized Cyclic AMPs^a^

a (A) US-SPPS-Fmoc deprotection: 20% piperidine in DMF, 0.5 + 1 min; (B) US-SPPS coupling: Fmoc-AA, COMU, OxymaPure, DIEA, DMF, 10 min. Path 1 (for peptides **2-10**): (c) Pd­(PPh_3_)_4_, NDMBA, DCM/DMF (3:2, v/v), 1 h × 2; (d) PyAOP, HOAt, DIEA, DCM/DMF (1:1, v/v), 12 h; (e) TFA/TIS/H_2_O (95:2.5:2.5, v/v/v), 3 h. Path 2 (for peptides **3-5**): (f) HgCl_2_, 1,3-bis­(*tert*-butoxycarbonyl)-2-methyl-2-thiopseudourea, DIEA, DMF, 16 h. Path 3 (for peptides **11-13**): (g) DPT, DCM, 12 h; (h) DCM/TIS/TFA (94:5:1, v/v/v), 20 min × 10; (i) TEA, THF, 12 h; (j) CH_3_I, DMF, 1 h × 3; (k) NH_4_OAc, NMM, DMSO, 80°, 12 h.

All peptides were cleaved from the resin and purified by reversed-phase high-pressure liquid chromatography (RP-HPLC). Each peptide was confirmed to possess >97% purity by analytical RP-HPLC, and their molecular masses were verified by ESI mass spectrometry (ESI-MS).

### Physicochemical Characterization

The key physicochemical parameters of the newly designed guanidino-based TL-derived peptides are reported in Table 1. Net charges ranged from +3 to +4. Both reference peptides (peptides **1** and **2**) carried a +3 charge, and this value was retained in derivatives featuring replacement of the *N*-terminal amino group (peptides **3–5**) or the Lys-to-Arg substitution (peptide **9**). By contrast, derivatives bearing an additional guanidino functionality, either as pendant group (peptides **6–8** and **10**) or as a bridging element (peptides **11–13**), reached a charge of +4. The proportion of nonpolar residues varied between 61.5 and 69.2%, with the parent peptides and bridged analogues retaining the higher values, including those in which the *N*-terminal amino group was replaced by a guanidino group. Pendant derivatives with extra positively charged functionalities (peptides **6–8** and **10**) instead showed a modest reduction, consistent with the introduction of polar groups and the resulting fine-tuning of amphipathic balance. Retention times determined by analytical HPLC supported these trends, as the more polar pendant derivatives eluted earlier (12.0–13.1 min), whereas the bridged analogues, likely more compact and hydrophobic, displayed longer retention times (up to 14.5 min). The theoretical molecular weights matched those determined by mass spectrometry. The reverse-phase high-performance liquid chromatography (RP-HPLC) chromatograms and mass spectra of these peptides are provided in the Supporting Information (Figures S1–S13), demonstrating that all peptides had a purity exceeding 97%. Another relevant physicochemical descriptor concerns the conformational behavior of the peptides in solution, which can be influenced by local chemical modifications and, in turn, affect their biological activity. Secondary structures predicted from CD analysis in aqueous solution highlighted the structural impact of these substitutions. Specifically, the reference peptides were largely unordered in solution, with minimal α-helix and predominant random coil, while pendant guanidino derivatives remained conformationally flexible, with low helical levels and a mixture of β-strand and random structures. In contrast, the bridged analogues, particularly peptides **11** and **12**, exhibited a marked stabilization of α-helical structures (76 and 63%, respectively), accompanied by a strong reduction in random coil, suggesting that conformational preorganization was strongly favored in this series.

### Antibacterial Activity

The antimicrobial efficacy of the TL_cyc_ analogues was assessed against a representative panel of Gram-positive and Gram-negative bacterial strains, by determining their minimal inhibitory concentrations (MIC), as summarized in Table 2. To provide an integrated measure of antimicrobial potency, we calculated the geometric mean (GM) of MIC values across all strains (GM_all_), as well as separately for Gram-positive (GM_Gram+_) and Gram-negative (GM_Gram–_) bacteria.

A general observation is that, although activity varied across Gram-positive bacterial strains depending on the specific analogue, all compounds exhibited improved activity against *E. coli* ATCC 25922 (MIC < 12.5 μM) compared to the reference peptide **1** (MIC, 25 μM). Against the other Gram-negative strains, *P. aeruginosa* ATCC 27853, *Acinetobacter baumannii* ATCC 19606, *E. coli* D21 and *P. aeruginosa* PAO1, the effects of the guanidino modifications were variable, with certain derivatives displaying enhanced activity and others retaining or only slightly reducing potency. Peptide **2**, designed based on previously reported SAR insights highlighting the beneficial role of stereoinversion at position 10 in enhancing antimicrobial activity and reducing cytotoxicity,^18^ indeed showed increased efficacy against both *E. coli* ATCC 25922 and *P. aeruginosa* ATCC 27853 relative to peptide **1**. Its activity against Gram-positive bacterial strains remained comparable, except for *S. aureus* and *Enterococcus faecalis*, where a 4-fold reduction in potency was observed (*S. aureus* MIC, 12.5 μM *vs* 3.12 μM for peptide **1**; *E. faecalis* MIC, 25 μM *vs* 6.25 μM for peptide **1**). Based on these results, the d-Glu residue at position 10 was retained in subsequent guanidino-functionalized derivatives.

All guanidino-modified analogues displayed broad and potent antimicrobial activity against Gram-positive bacteria, with MICs ranging from 0.39 to 25 μM, except for peptide **10**, which showed reduced efficacy against *S. aureus* (MIC, 100 μM) and *E. faecalis* (MIC, 50 μM). Within the “guanidino-pendant group” series (series A), peptide **4** was particularly notable, showing a 2-fold reduction in MICs for *Staphylococcus epidermidis* and *Streptococcus agalactiae* (MIC, 1.56 μM *vs* 3.12 μM for peptide **1**) and for *Bacillus megaterium* (MIC, 0.78 μM *vs* 1.56 μM for peptide **1**). Similarly, in the “guanidino-bridged group” series (series B), peptides **12** and **13** showed enhanced activity against *S. epidermidis* and *S. agalactiae* (MIC, 1.56 μM *vs* 3.12 μM for peptide **1**) and a 4-fold improvement against *B. megaterium* (MIC, 0.39 μM *vs* 1.56 μM for peptide **1**). In contrast, for *S. aureus* and *E. faecalis*, guanidino modification did not increase potency, and in some cases activity decreased substantially. Based on GM_Gram+_ values, peptides **4** and **5** were the only analogues showing lower values compared to peptide **1** (peptide **4**: GM_Gram+_ = 2.06; peptide **5**: GM_Gram+_ = 2.36 *vs* peptide **1**: GM_Gram+_ = 3.12), indicating an improved and broader spectrum of activity among Gram-positive pathogens.

The improvements were more pronounced against Gram-negative strains. In the series A (peptides **3–10**), analogues maintained or slightly reduced their potency against *A. baumannii* relative to peptide **1** (MICs 3.12 or 6.25 μM, *versus* 3.12 μM for peptide **1**), whereas in the series B (peptides **11–13**) MICs decreased to 1.56 μM, representing a 2-fold enhancement. Against *E. coli* ATCC 25922, both series showed substantial gains in potency, particularly peptides **4** and **7**, which displayed MICs of 3.12 μM, an 8-fold improvement over peptide **1** (MIC, 25 μM). These improvements were mirrored in the *E. coli* D21 mutant strain, for which most analogues exhibited reduced MICs. Notably, peptides **6**, **7** and **12**, achieved MICs of 3.12 μM, corresponding to a 4-fold improvement. Activity against *P. aeruginosa* was particularly relevant, given its high intrinsic resistance to conventional antibiotics. While peptides **3** and **9** were poorly active (MIC, 100 μM, comparable to peptide **1**), several analogues displayed markedly enhanced efficacy. Peptides **5**, **8**, **10**, **11**, and **13** had MICs of 50 μM, peptides **4** and **6** reached 25 μM, and the best performers were peptides **7** and **12**, with MICs of 12.5 μM, representing an 8-fold improvement over peptide **1**. This trend was confirmed for the more virulent *P. aeruginosa* PAO1 strain, where peptides **6**, **7**, **12** and **13** exhibited MICs between 6.25 and 12.5 μM, compared with 25 μM for peptide **1**. These data indicate that both pendant and bridged guanidino modifications significantly enhance performance even against virulent Gram-negative bacteria. The GM_Gram–_ values further highlight this improvement, indicating both increased potency and enhanced selectivity toward Gram-negative bacteria compared to peptide **1** (GM_Gram–_ = 18.9). All derivatives showed lower GM_Gram–_ values (<15 μM), with peptides **6**, **7** and **12** emerging as the most active (peptide **6**: GM_Gram–_ = 7.16; peptide **7**: GM_Gram–_ = 4.72; peptide **12**: GM_Gram–_ = 4.72).

### Cytotoxicity against Human Keratinocytes

Following the evaluation of antimicrobial activity, all TL_cyc_ analogues (peptides **2–13**) were evaluated in cytotoxicity assessment against human keratinocytes (HaCaT cells) after short- (2 h) and long-term (24 h) exposure. The results after 24 h are summarized in Table 3, while data from the 2 h assays are reported in the Supporting Information (Table S2). Overall, all peptides exhibited good tolerability at low concentrations, maintaining cell viability above 85% up to 12.5 μM. In this concentration range, no significant cytotoxic effects were observed, confirming the overall biocompatibility of both series at doses comparable to or even exceeding their antimicrobial MIC values. At 25 μM, mild cytotoxic effects began to appear for some derivatives. Peptides **4**, **5**, **11–13** showed a marked decrease in cell viability (ranging from 21 to 58%), while peptides **2**, **3**, **6**, and **7** maintained higher tolerance (85–94% viability). In contrast, peptides **8–10** exhibited a moderate increase in viability (>100%), likely reflecting minor assay variability or compensatory cellular responses at sublethal doses. As the concentration increased to 50 μM, most peptides showed a clear drop in viability, with several compounds (**3–5**, **7**, **11–13**) reducing cell survival below 30%, indicating strong dose-dependent toxicity. Peptides **2**, **6**, and **8**, however, retained relatively higher viability (>70%), showing a more favorable cytotoxicity profile. At 100 μM, all peptides induced near-complete cell death, consistent with a marked cytolytic effect at high concentrations, with the exception of peptides **8** and **10**, which retained substantial cell viability (58 and 95%, respectively) even at the highest dose. However, these two analogues also displayed relatively weak antimicrobial activity (see Table 2), indicating that their lower cytotoxicity is likely associated with reduced membrane-disruptive potential rather than improved selectivity.

The calculated CC_50_ values further support above observations. Peptides **2**, **6**, **8**, and **9** showed the highest CC_50_ values (>50 μM), indicating the lowest cytotoxicity, while peptides **4**, **11–13** exhibited CC_50_ values in the range of 19–21 μM, suggesting higher susceptibility of keratinocytes to these analogues. Peptides **3**, **5** and **7** displayed intermediate profiles (CC_50_ = 25–50 μM). Importantly, when comparing these data with the corresponding antibacterial potencies, the calculated therapeutic indices (*T*_index_ = CC_50_/GM_all_) ranged from 3.4 to 11.8, demonstrating that most peptides maintain an acceptable balance between antibacterial efficacy and cytotoxicity.

### Structure–Activity Relationship Analysis

As shown in Figure 3, the modification strategies at different molecular sites within the guanidino-based TL_cyc_-derived peptide analogues designed in this study, and their intended optimization purposes are summarized. Guanidino functionalities were introduced through two main strategies: as pendant groups attached to accessible residues, and as bridged groups incorporated within the cyclizing motif in place of the neutral lactam bridge. These two complementary approaches allowed us to explore how the spatial positioning of additional positive charges, either externally exposed or structurally embedded, could modulate physicochemical parameters and AMP performance. Analysis of the physicochemical parameters revealed distinct effects of these modifications: pendant guanidino groups primarily increased the net positive charge and polarity without enforcing a defined secondary structure, whereas guanidino-bridged architectures strongly stabilized helical conformations. The interplay between charge distribution, hydrophobicity, chromatographic retention, and secondary structure provides valuable insight into the subsequent biological behaviors of these analogues. The antimicrobial results demonstrated that guanidino substitution within the TL_cyc_ scaffold markedly modulated both potency and selectivity. When introduced as a pendant moiety at the *N*-terminal segment (Phe^1^-Pro^3^), a region considered outside the structured α-helix,^22^ the modification generally enhanced activity. Indeed, substitution of the *N*-terminal amino group with a guanidino function *via* β-alanine (peptide **4**) or GABA (peptide **5**), maintained or improved potency relative to peptide **1**, with notable gains against Gram-positive strains (peptide **4**: GM_Gram+_ = 2.06; peptide **5**: GM_Gram+_ = 2.36). In contrast, placement of the guanidino group on the *para*-position of Phe^1^ or at position 4 of the Pro^3^ ring (peptides **6** and **7**, respectively) resulted in reduced activity against *S. aureus* but enhanced efficacy toward Gram-negative bacteria (peptide **6**: GM_Gram–_ = 7.16; peptide **7**: GM_Gram–_ = 4.72), particularly peptide **7**, which was highly effective against *P. aeruginosa* ATCC 27853 (MIC, 12.5 μM) and *P. aeruginosa* PAO1 (MIC, 6.25 μM). Modification at Phe^5^ (peptide **8**) did not substantially alter activity. Based on peptide **1**, this *N*-terminal region (1–5) assumes a less-defined and flexible conformation, possibly involving an inverse γ-turn centered on Pro^3^.^22^ Within the amphipathic α-helical region (residues 5–13), introduction of the guanidino group at position 7 (Lys → Arg, peptide **9**) and position 8 (Phe → 4-guanidinophenylalanine, peptide **10**) appeared detrimental, possibly due to the disruption of the hydrophobic face of the helix. Conversely, affecting the chemical bridge by replacing the neutral amide with a positively charged guanidino group substantially enhanced antimicrobial properties. The resulting bridged analogues (peptides **11**-**13**) outperformed peptide **1** across nearly all tested strains, although peptides **12** and **13** showed lower potency against Gram-positive and Gram-negative (peptide **12**: GM_Gram+_ = 2.06 and GM_Gram–_ = 4.72; peptide **13**: GM_Gram+_ = 2.06 and GM_Gram–_ = 8.22). Interestingly, comparison of the MICs of analogues with a similar number of positive charges (*e*.*g*., compounds **6**–**8** and **10** *vs* **11**–**13**) indicated that the observed activity improvements are not solely attributable to charge magnitude but rather to the specific spatial positioning of guanidino functionalities within the sequence. Cytotoxicity data further supported this structure–activity relationship. Among derivatives, peptides **6–8** and **12** exhibited the most favorable safety profiles, showing CC_50_ values above their antimicrobial MICs and *T*_index_ values (≥6). Notably, peptides **7** and **12** combined potent antibacterial effects, particularly against *P. aeruginosa* and *E. coli*, with negligible cytotoxicity at therapeutically relevant concentrations (≤12.5 μM). In contrast, the bridged analogues (peptides **11–13**) tended to show delayed cytotoxicity effects at higher doses, possibly reflecting stronger but less selective interactions with eukaryotic membranes. Overall, all guanidino-functionalized TL_cyc_ analogues were well tolerated by keratinocytes within their effective antimicrobial concentration range.

**Figure 3:**
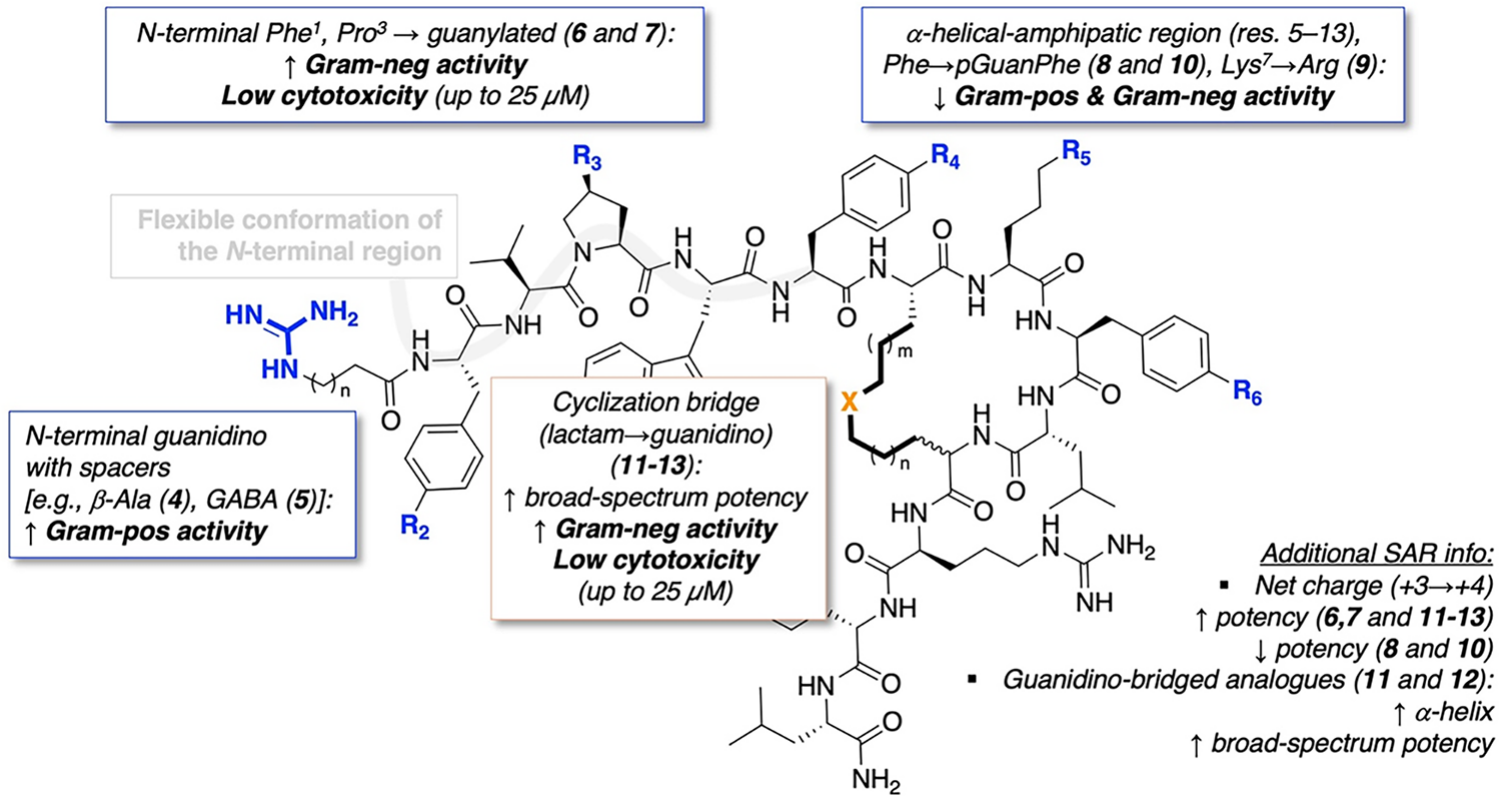
General chemical structure of the newly designed guanidino-based TL_cyc_-derived peptides highlighting the modified sites and their associated biological effects. R denotes positions modified with pendant guanidino groups, while X represents the guanidino bridge introduced in place of the lactam linkage. Boxes summarize the key outcomes of each modification strategy.

The combined analysis of physicochemical, antimicrobial, and cytotoxicity data highlights that the introduction of guanidino groups at synthetically accessible positions within the TL_cyc_ scaffold can markedly influence both potency and selectivity. A structure-activity-toxicity picture was drawn (Figure 3), and some consistent trends emerge regarding the impact of charge distribution and substitution site on biological behavior. Based on these results, peptides **2**, **6**, **7** and **12** were selected for subsequent investigations. Peptide **2** was included as an improved reference compound, combining the beneficial stereoinversion at position 10 with favorable balance between activity and cytocompatibility. Peptides **6** and **7**, belonging to the guanidino-pendant series, were chosen for their superior antibacterial profiles against Gram-negative strains along with negligible cytotoxicity up to 25 μM. Peptide **12**, representing the guanidino-bridged series, stood out for its potent and broad-spectrum activity coupled with an acceptable safety margin. Collectively, these peptides provide a representative and balanced set for subsequent studies aimed at characterizing their functional behavior.

### Hemolytic Activity

Given their membrane-active nature, peptides **2**, **6**, **7**, and **12** were further examined for potential off-target effects on mammalian cells. To complement keratinocyte cytotoxicity data and better assess membrane selectivity, additional assays were performed on BEAS-2B cells (see Supporting Information, Table S3) and on red blood cells (RBCs) to evaluate hemolytic potential (Figure 4). All peptides exhibited negligible cytotoxicity at low concentrations, consistent with the results obtained in HaCaT cells. However, a moderate decrease in BEAS-2B viability was observed at higher doses, particularly for peptides **2** and **6** at 50 μM, and for **7** and **12** starting at 25 μM. Notably, cell viability remained acceptable, ∼85% for peptide **7** and ∼60% for peptide **12**, even at concentrations corresponding to their antipseudomonal MIC values (12.5 μM, see Table 2). A similar concentration-dependent result was observed in hemolytic assays (Figure 4). All tested peptides caused limited RBC lysis at low concentrations, with hemolysis ≤ 50% at 12.5 μM. Among them, peptide **6** displayed the lowest hemolytic activity across the tested range, whereas peptide **12** showed slightly higher lytic potential at the upper concentrations (25–50 μM).

**Figure 4:**
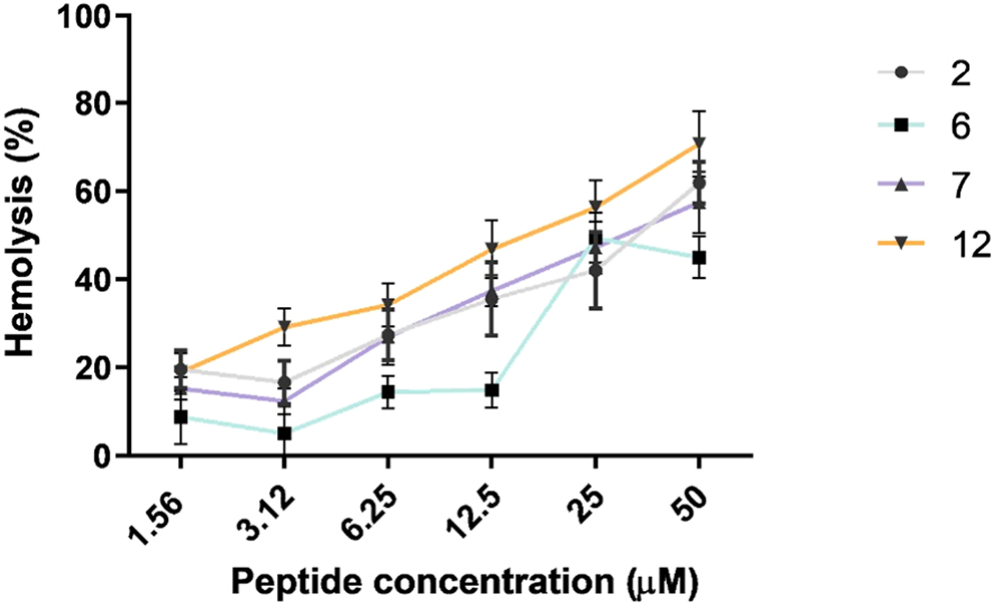
Effect of peptides **2**, **6**, **7** and **12** on mammalian RBCs after 30 min of treatment at 37 °C. The percentage of hemolysis was calculated with respect to the control (cells treated with vehicle). Data are the means ± standard deviation (SD) of three independent experiments.

### Antibiofilm Activity and Fluorescence Microscopy Analysis

Many pathogenic bacteria readily colonize biotic and abiotic surfaces, forming sessile communities known as biofilms. These structures are characterized by bacterial populations embedded in an extracellular matrix, which provides physical protection against antibiotic agents and contributes to the emergence of resistant and persistent infections. Therefore, antimicrobial compounds capable of targeting biofilm-associated bacteria are of particular interest. To assess this property, the selected peptides (*i*.*e*., **2**, **6**, **7**, and **12**) were tested evaluating the biofilm eradication potency against two clinically relevant human pathogens such as the Gram-positive *S. aureus* ATCC 25923 and the Gram-negative *P. aeruginosa* ATCC 27853 (Figure 5). Against *S. aureus* biofilms, all peptides exhibited potent antibiofilm activity at 50 μM, with a reduction in biofilm viability exceeding 90%. Notably, peptides **7** and **12** maintained significant activity at lower concentrations (below 12.5 μM), achieving approximately 50% reduction in biofilm viability even at 6.25 μM. In contrast, peptides **2** and **6** demonstrated weaker or no activity at concentrations below 25 μM, consistent with their lower antimicrobial potency (see Table 2). Against *P. aeruginosa* biofilms, the peptides exhibited overall reduced activity compared to *S. aureus*. Peptides **7** and **12** showed the most pronounced antibiofilm effects, with significant viability reductions (over 50%) at 25 and 50 μM, whereas peptide **2** was largely inactive across all tested concentrations (>70% biofilm viability). This reduced activity against *P. aeruginosa* biofilms likely reflects the increased structural complexity and intrinsic resistance mechanisms of Gram-negative biofilms.

**Figure 5:**
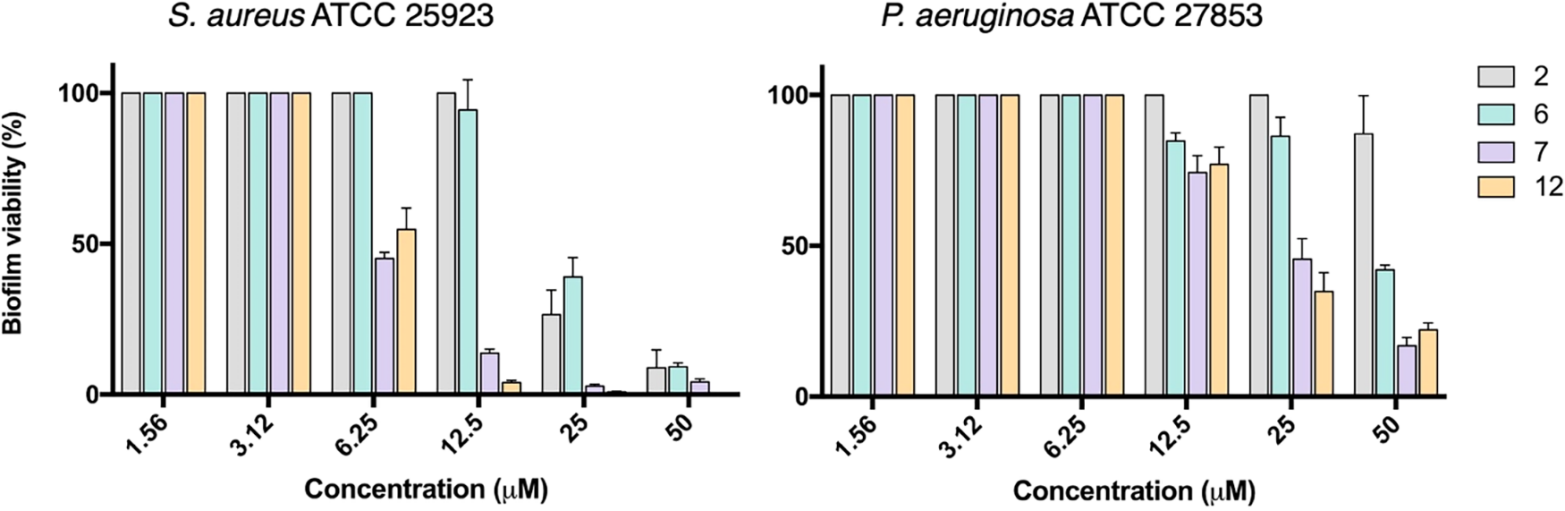
Activity of peptides **2**, **6**, **7** and **12** against preformed biofilms of *S. aureus* ATCC 25923 and *P. aeruginosa* ATCC 27853. Biofilm viability was assessed by quantifying the reduction of MTT to its insoluble formazan product and is expressed as a percentage relative to untreated control samples (set at 100% viability). Data represent the mean ± SEM from three independent experiments.

These findings highlight the potential of selected peptides, particularly **7** and **12**, as promising candidates for the treatment of biofilm-associated infections. The ability to retain antibiofilm activity at relatively low concentrations is especially relevant for addressing chronic infections where biofilm formation plays a central role in therapeutic failure.

To further examine peptide effects on preformed sessile communities, representative peptides **7** and **12** were analyzed by fluorescence microscopy under the same previously described assay conditions. Biofilms of *S. aureus* and *P. aeruginosa* were treated with each peptide at concentrations corresponding to their MIC and 4 × MIC (6.25 and 25 μM for *S. aureus*; 12.5 and 50 μM for *P. aeruginosa*). Following treatment, cells were stained with SYTO 9 and propidium iodide (PI) to visualize live and dead cells, respectively (Figure 6). SYTO 9 permeates all cells and fluoresces green upon binding nucleic acids, whereas PI penetrates only cells with compromised membranes, emitting red fluorescence.^35^

**Figure 6:**
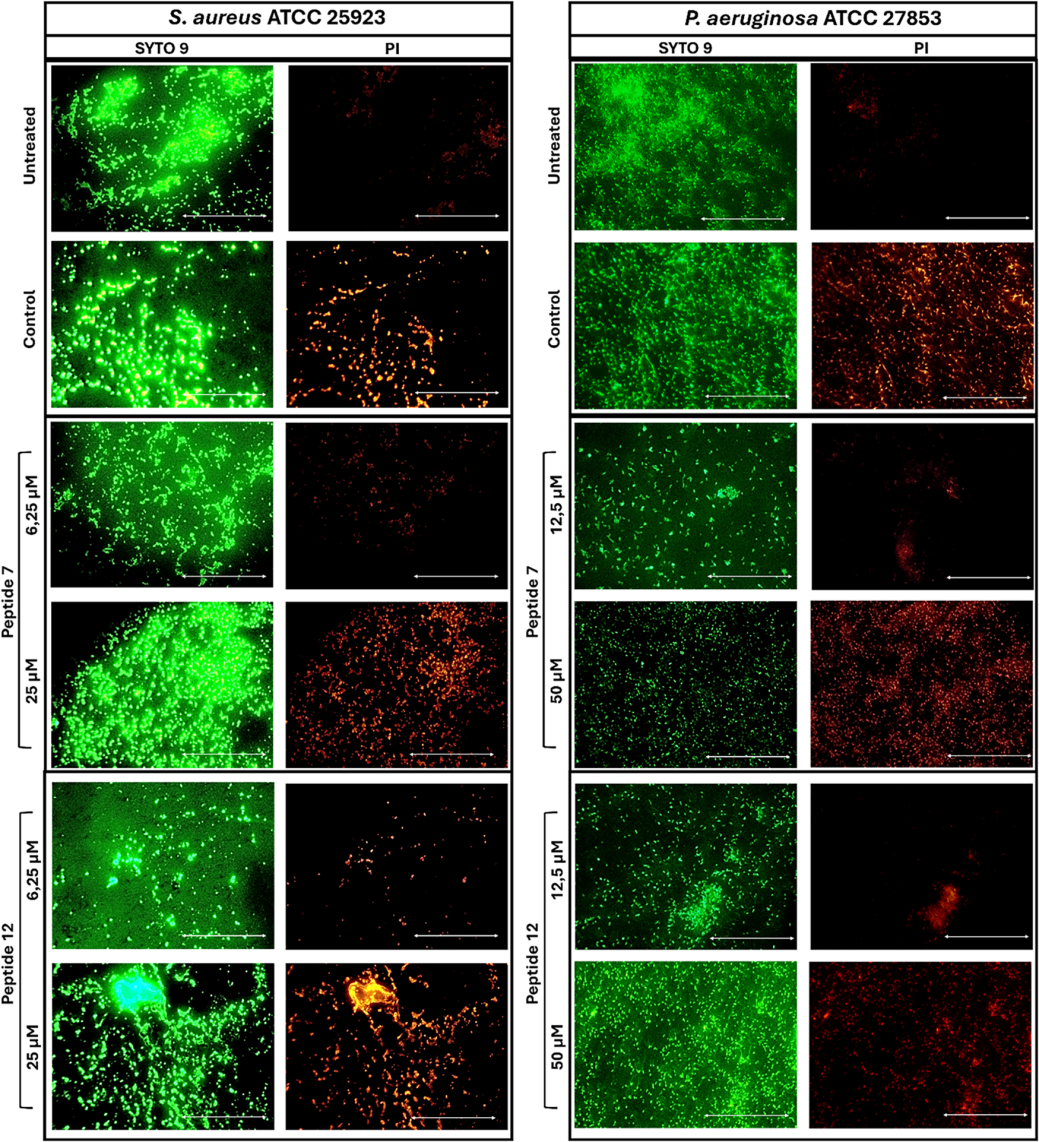
Fluorescence microscopy images of *S. aureus* ATCC 25923 (left) and *P. aeruginosa* ATCC 27853 (right) biofilms exposed for 2 h to peptides **7** and **12** at MIC and 4 × MIC concentrations (6.25 and 25 μM for *S. aureus*; 12.5 and 50 μM for *P. aeruginosa*). Biofilms were stained with SYTO 9 and PI. Control is given by biofilms treated with 70% ethanol to provoke cell death. Scale bars (white arrow) correspond to 100 μm.

Fluorescence microscopy images revealed a marked increase in red fluorescence (dead cells) in biofilms treated with ethanol and the highest peptide concentrations, while untreated or low-dose samples predominantly displayed green fluorescence, indicative of viable cells. In contrast, only a small number of dead cells were observed in samples exposed to the lower peptide concentrations and in the untreated controls. These observations are fully consistent with the quantitative biofilm assays (Figure 5), confirming the concentration-dependent antibiofilm activity of the tested peptides and reinforcing peptides **7** and **12** as promising candidates for combating biofilm-associated infections.

### Bacterial Membrane Interaction Studies

Cyclic temporins are known to exert their antimicrobial activity primarily through interactions with bacterial membranes.^22^ Their main mechanism of action (MOA) involves membrane disruption, either through a carpet-like mechanism or by forming pores.^22,36^ Given this, we investigated the MOA of selected analogs (peptides **2**, **6**, **7** and **12**) exhibiting the most promising biological profiles, in terms of both antimicrobial/antibiofilm activities and low cytotoxicity, employing fluorescence-based biophysical assays on model membranes, alongside *in vitro* assays on bacterial cells.

#### Assessment of Peptides’ Insertion and Interaction with Model Membranes

Previous biophysical investigations using liposome-based models mimicking Gram-positive and Gram-negative bacterial membranes, supported for peptide **1** a membranolytic mechanism characterized by liposome disruption *via* pore formation.^22^ Building on this, we analyzed the membrane binding and insertion behavior of cyclic derivatives **2**, **6**, **7** and **12**. The study exploited the fluorescence emission properties of the Trp residue, located at position 4 in all peptides. Trp fluorescence emission changes in a hydrophobic environment and its insertion into lipid bilayer was monitored through quenching experiments using acrylamide, a small, water-soluble quencher unable to penetrate lipid bilayers.^37^ When Trp residues are exposed to the aqueous environment, acrylamide can quench their fluorescence, leading to a decrease in the Trp quantum yield recorded. Trp quenching assays were performed in water and large unilamellar vesicles (LUVs) composed of DOPC:CL (58:42, mol/mol) to mimic Gram-positive membranes, and DOPE:DOPG:CL (65:23:12, mol/mol/mol) to model Gram-negative membranes. As shown in Figure 7A, in aqueous solution Trp residues of all peptides were fully accessible, as indicated by a progressive decrease in fluorescence emission upon acrylamide addition (concentrations ranging from 0.02 to 0.22 M). The degree of Trp accessibility to acrylamide was determined by the Stern–Volmer quenching constant (*K*_sv_), calculated through an analysis of the quenching data with the Stern–Volmer eq (Figure 7B): *F*_0_/*F* = 1 + *K*_sv_ [Q], where *F*_0_ and *F* represent the fluorescence intensities before and after the addition of the quencher (Q), respectively. The high *K*_sv_ values obtained in solution confirmed complete Trp accessibility for all peptides (Figure 7C).

**Figure 7:**
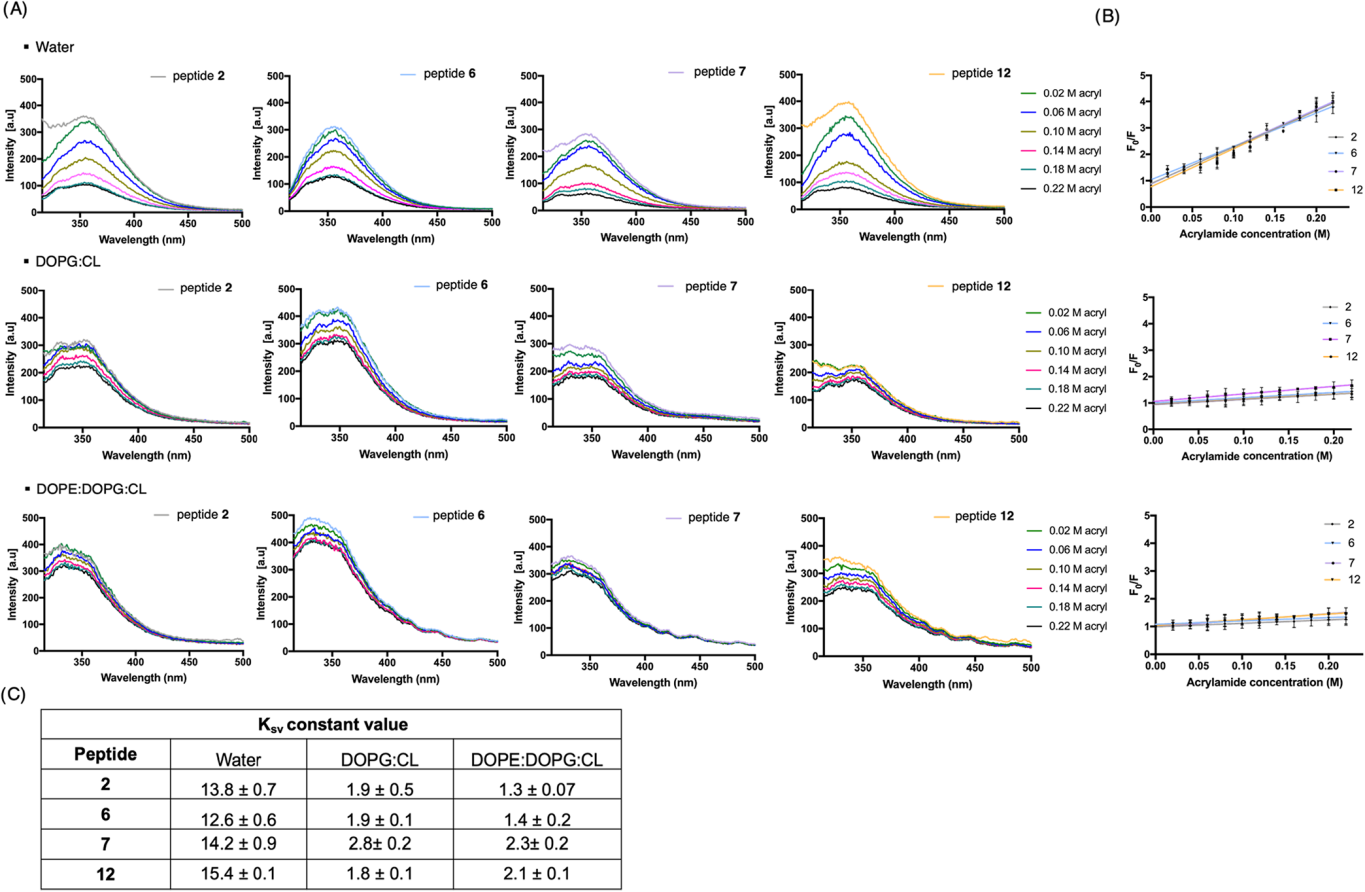
Tryptophan fluorescence quenching of peptides by acrylamide in different environments. Panel A shows fluorescence emission spectra of peptides **2**, **6**, **7**, and **12** upon titration with increasing concentrations of acrylamide (0.02 to 0.22 M) in water, in the presence of LUVs composed of DOPG:CL (mimicking Gram-positive bacterial membranes), and in the presence of LUVs composed of DOPE:DOPG:CL (mimicking Gram-negative bacterial membranes). Panel B displays Stern–Volmer plots, while panel C summarizes the corresponding Stern–Volmer quenching constants (*K*_sv_).

To monitor the interaction and incorporation of each peptide into the membrane, experiments were also performed using LUVs. As shown in Figure 7A, in the presence of LUVs mimicking Gram-positive and Gram-negative membranes, all peptides exhibited a reduced quenching of Trp fluorescence compared to that observed in aqueous solution, indicating only a partial accessibility of Trp to the quencher and the insertion of the Trp of all peptides into the lipid bilayer. Consistently, the *K*_sv_ values calculated in the presence of LUVs were lower than those obtained in aqueous solution (Figure 7C). The decreased *K*_sv_ values reflect the reduced accessibility of the Trp residues to acrylamide, as they become partially shielded within the hydrophobic environment of the LUVs. Based on these results, all peptides exhibited strong affinity and interaction with bacterial membranes, driven by multiple factors such as their net positive charge and the balance between hydrophilic and hydrophobic regions. In this study, the introduction of a guanidino group either as a pendant in peptides **6** and **7** or as a bridge in peptide **12** led to a slight increase in the net positive charge from +3 (as in peptide **2**) to +4, but without changing their behavior to insert into lipid bilayer which is also influenced by other physicochemical properties. Indeed, while the net positive charge is implicated in electrostatic interactions with the anionic surface of liposomes, the hydrophobic region represented by aromatic residues (*e*.*g*., Phe, Trp) plays a crucial role in promoting membrane insertion.^38^ In this context, our findings obtained on Trp quenching revealed a strong ability of all peptides to incorporate into the lipid bilayer which is further supported by additional quenching experiments using bromo probes bound to the lipid chains of the membrane (Figure 8). Specifically, two different phospholipids containing bromo at distinct positions were employed: 1-palmitoyl-2-(4,5-dibromo)­stearoyl-*sn*-glycero-3-phosphocholine (4,5-Br-PC), where bromo is located at the interface of LUVs, and 1-palmitoyl-2-(11,12-dibromo)­stearoyl-*sn*-glycero-3-phosphocholine (11,12-Br-PC), where bromo is located inside their lipid core. LUVs mimicking Gram-positive and Gram-negative membranes were prepared by incorporating 25% of each bromo-bound phospholipid.

**Figure 8:**
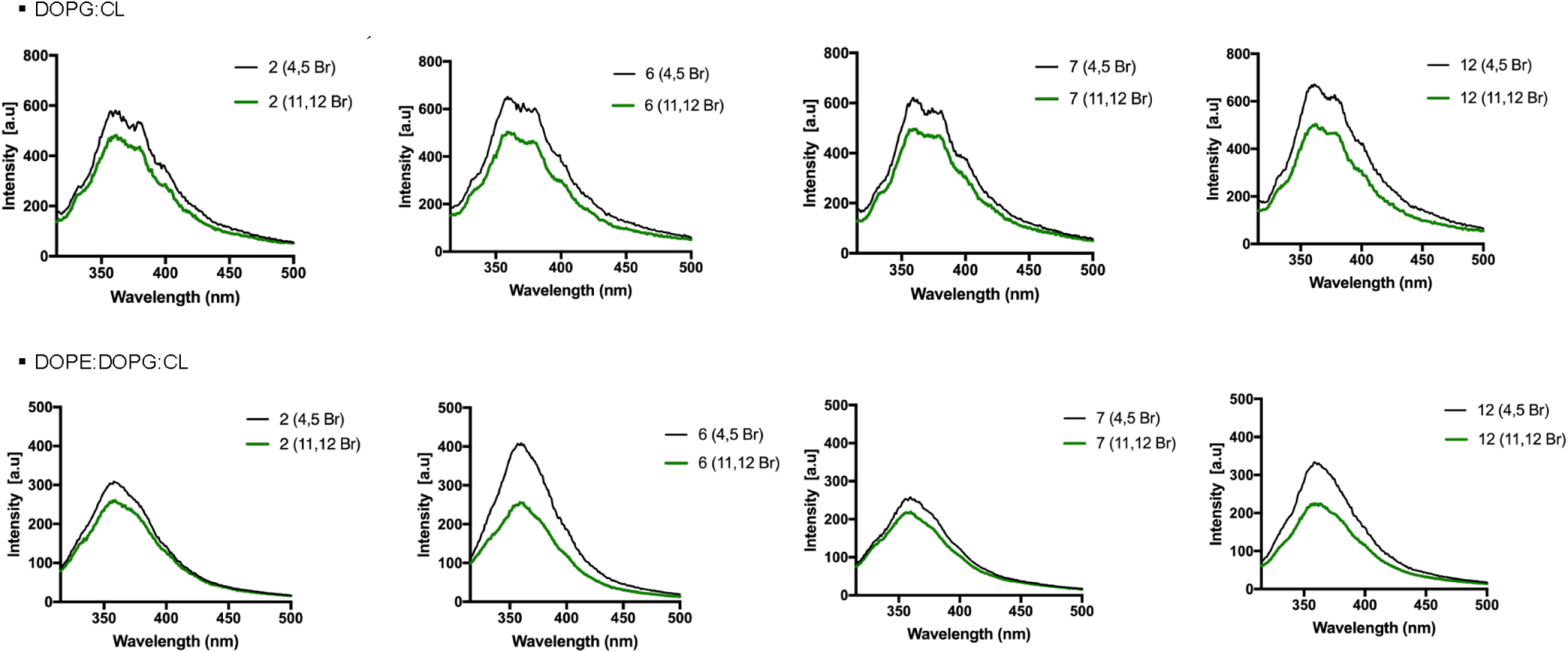
Tryptophan fluorescence spectra for each peptides **2**, **6**, **7** and **12**, recorded in LUVs made of DOPG:CL (Gram-positive) and DOPE:DOPG:CL (Gram-negative) bearing the probes 4,5-Br-PC and 11,12-Br-PC.

Following the addition of each peptide (10 μM) to the LUVs, Trp spectra were recorded upon excitation at 295 nm. As observed in Figure 8, the largest quenching of Trp was recorded in the presence of 11,12-Br-PC for all peptides, while it was less in the presence of LUVs made of 4,5-Br-PC where the quencher is located at the interface. These results confirmed that all peptides are deeply buried inside the lipid bilayer with the Trp residue oriented toward the lipid core.

#### Peptides’ Oligomerization and Its Impact on Membrane Fluidity

While self-aggregation in solution can hinder the antimicrobial activity of AMPs, oligomerization within bacterial membranes is crucial for their mechanism of action.^39^ To investigate this, we monitored peptides’ oligomerization in LUVs mimicking Gram-positive and Gram-negative membranes by using Thioflavin T (ThT), a fluorescent probe that increases its emission at 482 nm in hydrophobic environments such as peptide aggregates. In LUVs mimicking the Gram-positive membrane, all peptides showed the ability to oligomerize, though with different trends (Figure 9). Peptides **2** and **6** displayed the strongest capability to oligomerize reaching a plateau at the peptide/lipid (P/L) ratio of 0.2. Peptide **7** showed a concentration-dependent increase in ThT fluorescence, indicating progressive oligomerization, with a large ThT enhancement at the P/L ratio of 0.5. In contrast, peptide **12** exhibited minimal oligomerization, reaching ∼25% aggregation at the highest P/L ratio of 0.5.

**Figure 9:**
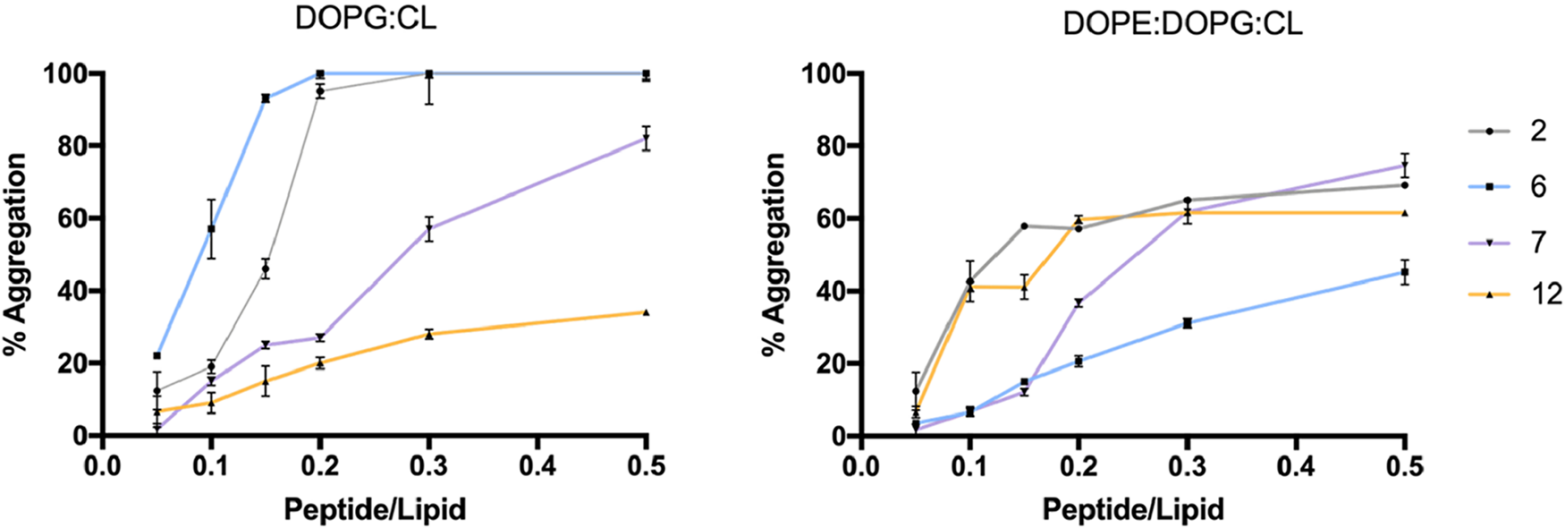
Peptide aggregation in LUVs mimicking Gram-positive (DOPG:CL) and Gram-negative (DOPE:DOPG:CL) membranes monitored by recording ThT fluorescence.

Remarkably, in LUVs mimicking Gram-negative membrane (Figure 9), all peptides demonstrated a progressive oligomerization profile. Peptides **2**, **7**, and **12** reached approximately 60% aggregation at the highest peptide-to-lipid (P/L) ratio of 0.5, whereas peptide **6** displayed a lower tendency to oligomerize, achieving only around 40% aggregation at the same P/L ratio. These results indicate a clear propensity of the peptides to assemble within the membrane environment, which may impact the membrane physical properties. To assess this potential effect, we evaluated membrane fluidity using the Laurdan assay. Laurdan is a fluorescent probe that changes its emission spectrum when the phospholipid state changes switching from an ordered state with a maximum at 440 nm in a disordered state with a maximum at 490 nm. The influence on the membrane fluidity was measured by calculating the generalized polarization (GP) value at the peptide concentrations of 25 and 50 μM (Table S4, Supporting Information). Before peptide addition, both Gram-positive and Gram-negative LUVs were in a disordered phase with a maximum emission at 490 nm. At 25 μM, no significant change in GP was observed, indicating that membrane fluidity remained largely in a disordered state. However, at 50 μM a notable shift toward more ordered membrane states was detected for all peptides, with negative GP values becoming less negative or even positive. Interestingly, all peptides strongly affected the DOPG:CL bilayers, inducing a transition to a more ordered phase (maximum at 440 nm). This is in line with their antimicrobial profile against Gram-positive strains, where all peptides exhibited a good activity such as the reference peptide **2**. A similar behavior was observed in the presence of the DOPE:DOPG:CL LUVs, where all peptides **2**, **6**, **7**, and **12** caused a shift of the GP value toward a more ordered state as reflected by positive GP values (Table S4). This result showed a significant effect on the membrane fluidity by all peptides in both conditions.

#### Structural Changes in the Presence of Model Membranes

The strong interaction between AMPs and membrane can induce secondary structural changes that can enhance their ability to induce a more efficient membrane leakage.^40^ We investigated the secondary structure of all peptides in water and LUVs by performing Circular Dichroism (CD) spectroscopy. In water, peptides **6** and **7**, bearing guanidino-pendants, exhibited a random coil structure, similar to the reference peptide **2** (Figure 10). Interestingly, peptide **12**, featuring a guanidino bridge, displayed a strong tendency to adopt an α-helical conformation, characterized by two minima near 208 and 222 nm.

**Figure 10:**
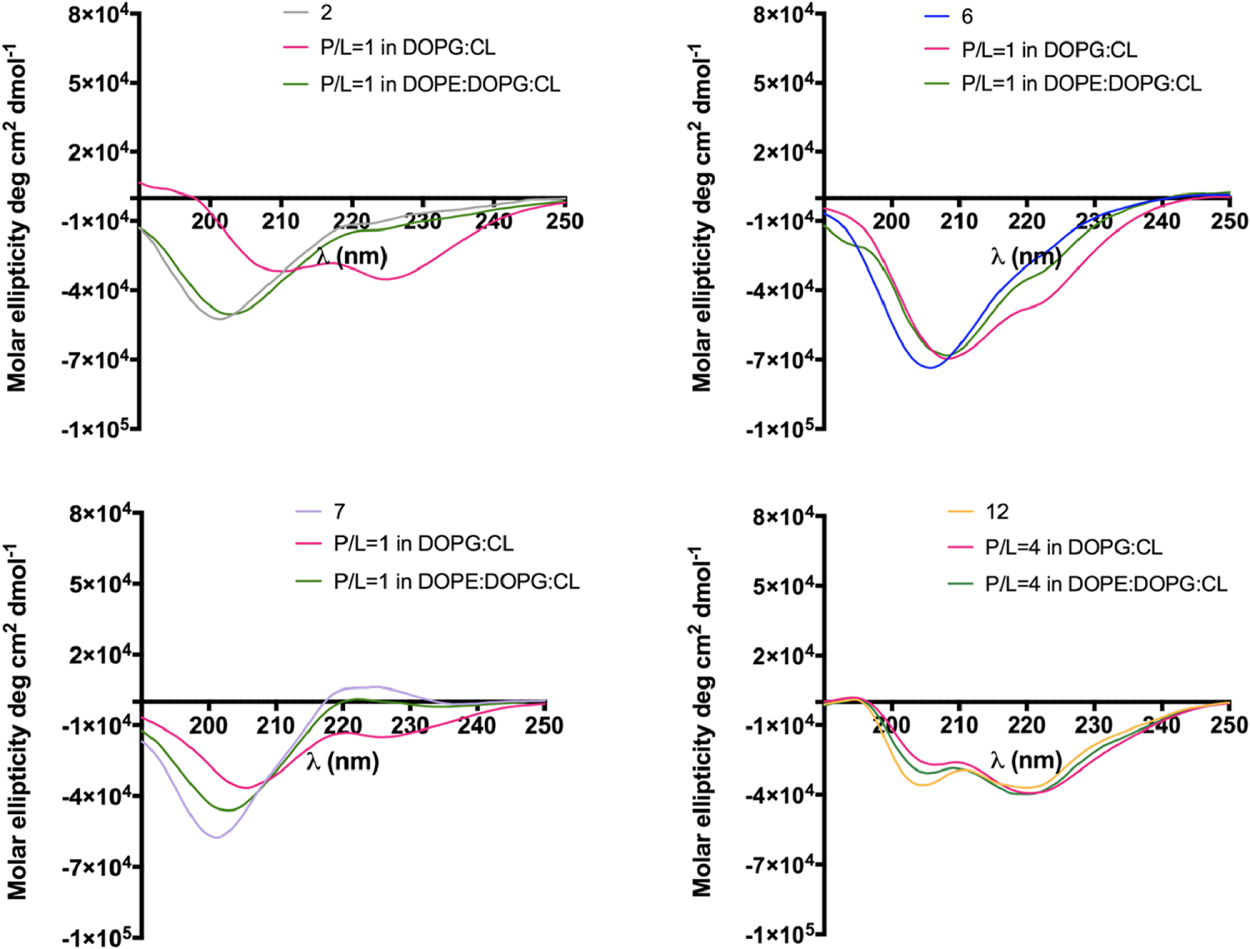
CD spectra of peptides **2**, **6**, **7** and **12** recorded in solution and in LUVs mimicking bacterial membranes.

In the presence of LUVs mimicking bacterial membranes, we used a P/L ratio of 1 for peptides **2**, **6** and **7**, while for peptide **12** the P/L ratio of 4 was used due to the strong propensity of the peptide to aggregate in liposomes. As observed in Figure 10, all peptides showed notable conformational changes upon the interaction with lipid bilayer. In particular, peptide **2** showed a propensity to adopt the helical structure in DOPG:CL but remained unstructured in DOPE:DOPG:CL. A similar behavior was observed for peptide **7** carrying the pendant group on Pro^3^ that changes its secondary structure adopting a slight helix in DOPG:CL and remained random coil in DOPE:DOPG:CL. In contrast to peptide **7**, peptide **6** with the guanidino-pendant on Phe^1^ showed a greater tendency to undergo conformational changes upon interaction with LUVs, adopting a partial helical structure under both membrane conditions. Notably, the incorporation of a guanidino bridge in peptide **12** promotes an α-helical conformation already in aqueous solution, which is maintained upon interaction with liposomes. As observed by CD spectra, the peptide **12** showed a marked tendency to form helical aggregates in LUVs, also confirmed by ratio of ellipticities at 222 and 208 nm (θ_222_/θ_208_). In both membrane models, the ratio θ_222_/θ_208_ exceeds 1 (θ_222_/θ_208_ = 1.5 for DOPG:CL; θ_222_/θ_208_ = 1.3 for DOPE:DOPG:CL) indicating the presence of the oligomeric helical structures since values below 1 are typically associated with monomeric helices.^41^

#### Membrane Fusion, Damage and/or Perturbation

We demonstrated that peptides are capable of interacting with and inserting into lipid bilayers, where they oligomerize and modulate membrane fluidity. To further explore their membrane-disruptive potential, we evaluated their ability to induce membrane fusion and leakage, which are two key hallmarks of AMP activity.

Membrane fusion, which may occur *via* pore formation or a carpet-like disruption mode, was assessed using a Förster resonance energy transfer (FRET).^42^ This assay uses nitrobenzodiazole (NBD) and rhodamine (Rho) as a pair of fluorophores. Both fluorophores, bound to the lipid phosphatidylethanolamine, were used to prepare labeled LUVs mimicking Gram-positive and Gram-negative membranes that were mixed with unlabeled LUVs. In this assay, LUVs made of DOPG:CL and DOPE:DOPG:CL were treated with increasing concentrations of each peptide at P/L ratios of 0.01, 0.03, 0.05, 0.1, 0.2, 0.3, and 0.5. All peptides caused membrane fusion of LUVs in a dose-dependent manner, observed as a decrease of NBD and an increase of Rho fluorescence intensities consistent with an increase in FRET efficiency. As shown in Figure 11, peptides **6**, **7** and **12** showed a strong capability to cause the fusion of LUVs made of DOPG:CL reaching the membrane saturation at the P/L ratio of 0.2. Peptide **2** also showed a fusogenic activity in a dose-dependent manner causing the highest percentage (100%) at the higher P/L ratio of 0.3. In the presence of LUVs made of DOPG:DOPE:CL, peptide behavior was slightly different, but the vesicle fusion was still measured (Figure 11). Peptides **2** and **12** induced moderate membrane fusion reaching 75% of fusion at the highest P/L ratio of 0.5, while peptides **6** and **7** caused a significant fusion already at lower P/L ratios before reaching the membrane saturation at the P/L of 0.5.

**Figure 11:**
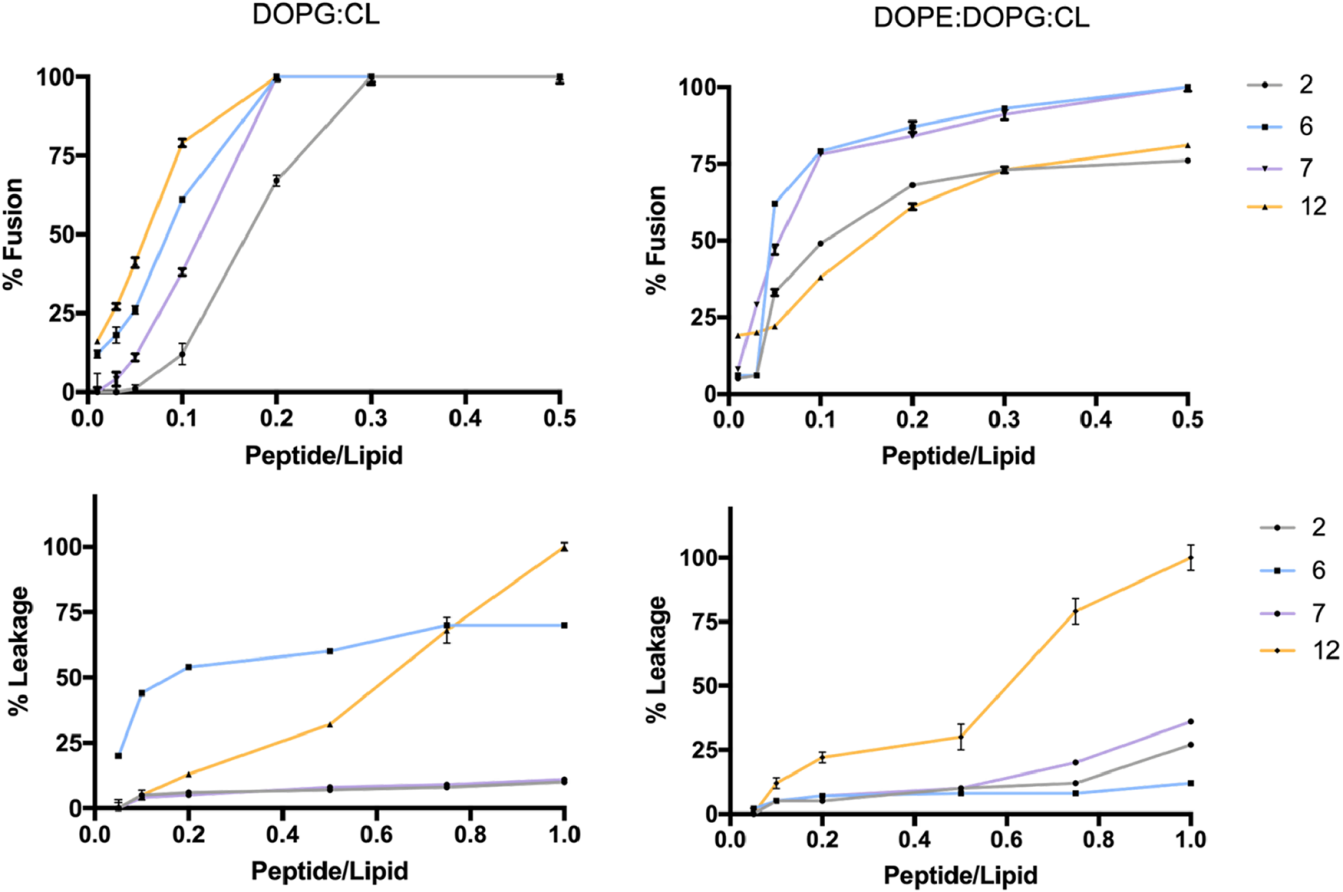
Fusion and leakage ability of peptides **2**, **6**, **7** and **12** on LUVs mimicking Gram-positive (DOPG:CL) and Gram-negative (DOPE:DOPG:CL) membranes.

In addition, the ability of all peptides to induce membrane damage through a carpet-like mechanism or pore formation was evaluated by performing the ANTS/DPX leakage assay. LUVs containing the fluorophore ANTS and its quencher DPX were exposed to increasing concentrations of peptides. Upon peptide-induced disruption, ANTS is released into the external medium, resulting in fluorescence emission at 512 nm. Interestingly, both in the presence of LUVs mimicking Gram-positive and Gram-negative membranes, we observed that peptide **12** had the strongest ability to induce membrane leakage causing 100% of liposome disruption at the highest P/L ratio of 1 (Figure 11). These findings suggest that its strong pore-forming ability may be attributed to the high helical content observed in LUVs, as revealed by CD measurements.

Regarding peptides **2** and **7**, they showed a low leakage effect on LUVs mimicking Gram-positive and Gram-negative membranes. In contrast, peptide **6** showed selective activity, inducing significant leakage only of Gram-positive LUVs but had no leakage effects on Gram-negative LUVs.

Overall, fusion and leakage results demonstrated the ability of each peptide to induce membrane damage, albeit through different mechanisms, depending probably on several factors, including the physicochemical properties and the secondary structure, which significantly influence the mechanism of action of AMPs. Our results suggest that peptide **12** may operate *via* a toroidal or barrel-stave mechanism, consistent with its strong membrane-disruptive activity, whereas the other peptides appear to act through a carpet-like mechanism driven by strong surface interactions.

#### Peptide Interaction and Permeability in Bacterial Cells

To validate the membrane activity of all peptides observed in liposome models and various fluorescence-based assays, a Sytox-based permeabilization assay was performed directly on bacterial cells. In this experiment, the fluorescent probe Sytox Green, whose fluorescence increases with the binding to nucleic acids and is impermeable to cells with an intact membrane, was used. The addition of an AMP can cause the membrane disruption and the consequent binding of the probe to nucleic acids with an increase in fluorescence signals. As reported in Figure 12A, against *S. aureus* peptides showed a dose-dependent increase in fluorescence signals from the addition of the peptide (time 0), with a lower effect for peptide **6**, consistent with its lower activity against this strain (MIC = 25 μM, see Table 2). Similar results were obtained against *P. aeruginosa* (Figure 12B). A dose-dependent perturbing effect was observed after peptide addition (time 0), with a lower intensity for peptide **2**, consistent with its lower antimicrobial activity (MIC = 50 μM) (Table 2). Representative fluorescence images of membrane-compromised cells after peptide **12** treatment were recorded with an Olympus optical microscope (×40) (Figure S15, Supporting Information).

**Figure 12:**
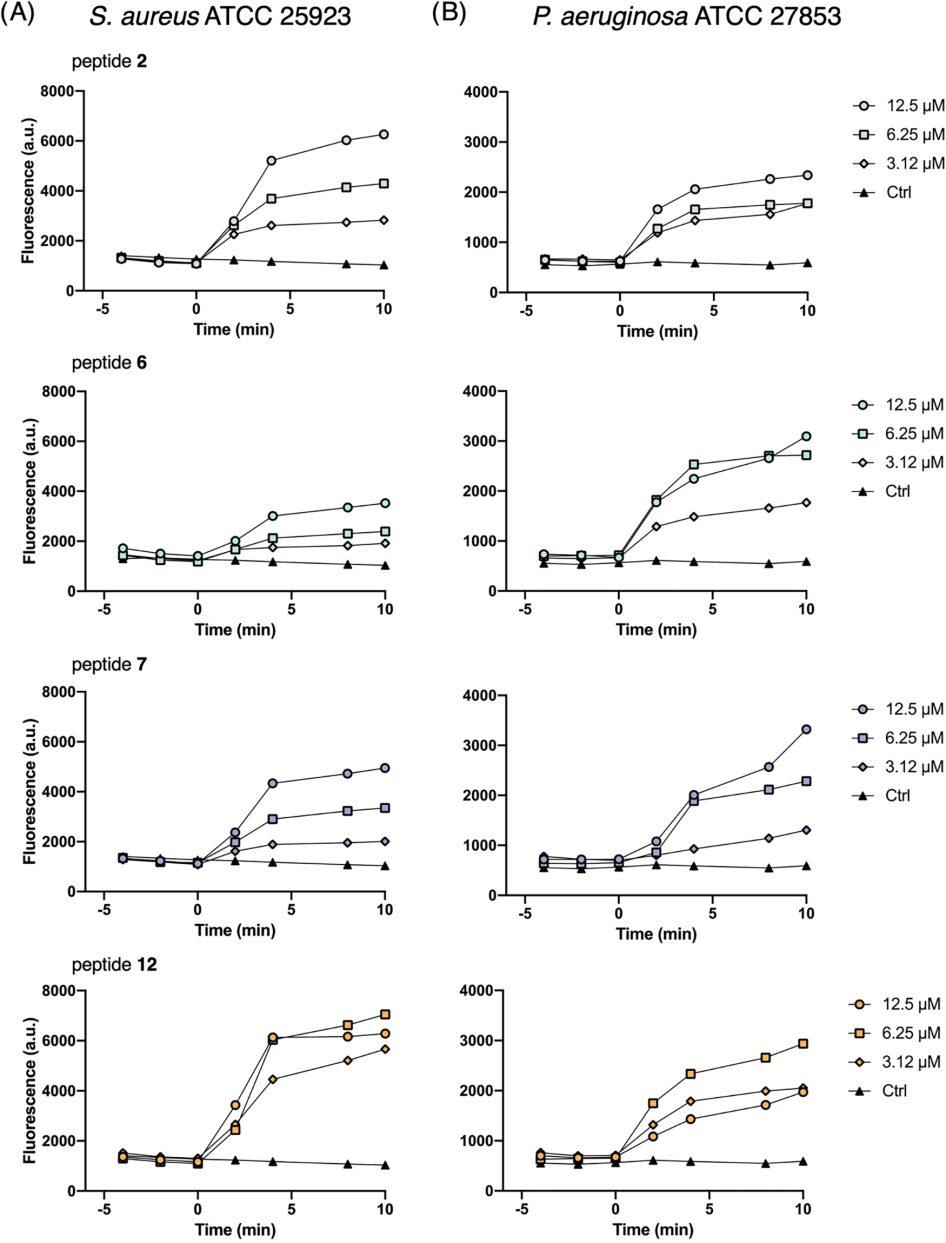
Kinetics of cytoplasmic membrane permeabilization of *S. aureus* ATCC 25923 (A) and *P. aeruginosa* ATCC 27853 (B) induced by the addition of peptides **2**, **6**, **7** and **12** at different concentrations. Controls (Ctrl) are untreated microbial cells. The reported values are from one representative experiment out of three.

### Killing Kinetics Assay

The fast membrane-perturbing action of the designed peptides was in line with their fast microbicidal activity that was investigated for the most active peptides (*i*.*e*., **7** and **12**) against both *S. aureus* ATCC 25923 and *P. aeruginosa* ATCC 27853 (Figure 13). As shown in Figure 13, at the highest concentration tested (2 × MIC), peptide **12** exhibited rapid bactericidal activity against both strains. Within 5 min, it caused a reduction of approximately 4-log units in viable cells of *S. aureus* and about a 2-log reduction for *P. aeruginosa*. Complete bacterial killing was achieved after 30 min at this concentration for both strains. Peptide **7** showed weaker microbicidal activity, with more than 3-log reduction in viable cells after 120 min at 2 × MIC.

**Figure 13:**
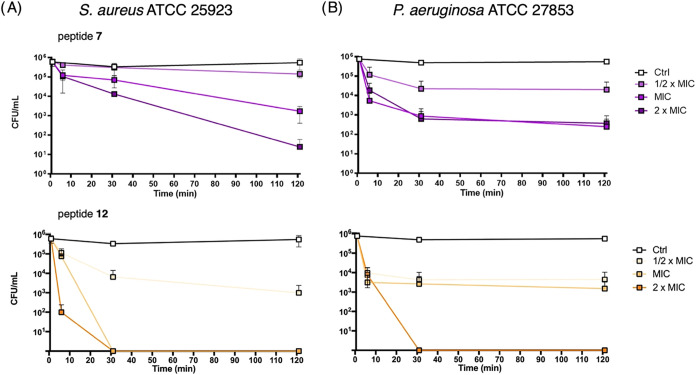
Effect of peptide **7** and **12** on *S. aureus* ATCC 25923 (A) and *P. aeruginosa* ATCC 27853 (B) cell viability. Bacteria (1 × 10^6^ CFU/mL) were incubated with both peptides at 2 × MIC, MIC, 1/2 × MIC, in phosphate-buffered saline (PBS) at 37 °C. The number of surviving cells (CFU/mL) was calculated at different time points (5, 30, and 120 min) and are reported as mean ± SD of two independent experiments.

### Proteolytic Stability

The stability of the selected peptide analogs (**2**, **6**, **7** and **12**) was evaluated in human serum and in the presence of the serine proteases trypsin and chymotrypsin, which preferentially cleave peptide bonds following basic or aromatic/hydrophobic residues, respectively.^43,44^ For serum stability assessment, each peptide was incubated in 90% human serum at 37 ± 1 °C, and analyzed over a 24 h period (Figure 14A). The percentage of intact peptide at different time points (0, 1, 2, 4, 8, and 24 h) was determined by calculating the peak area from HPLC chromatograms, as elsewhere reported.^45,46^ When compared with the degradation profile of peptide **1** reported previously,^22^ peptide **2**, identical except for the stereoinversion of Glu^10^, displayed a similar or slightly reduced stability, suggesting that this structural modification alone does not significantly improve protease resistance. When positioned at the *para* position of Phe^1^ (peptide **6**), the guanidino substitution resulted in an enhanced stability, particularly within the first 4 h of incubation, whereas insertion at position 4 of Pro (peptide **7**) led to a clear decrease in stability. Peptide **12** exhibited markedly improved stability, retaining over 50% integrity at 4 h and approximately 25% after 8 h.

**Figure 14:**
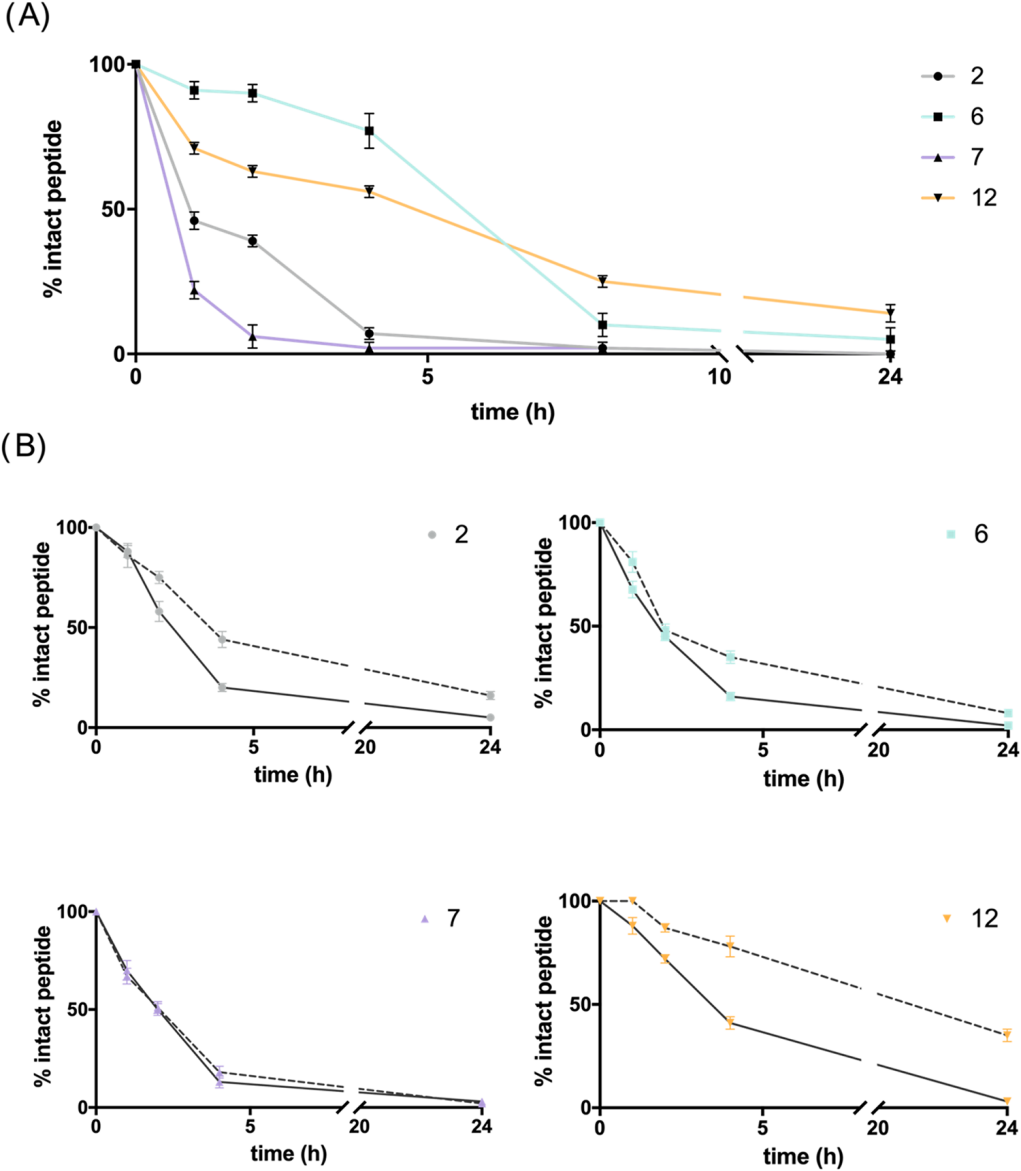
Proteolytic stability of **2**, **6**, **7** and **12** in human serum (A), and after the incubation with trypsin (dashed line) and chymotrypsin (solid line) (B) at different time intervals. Error bars are representative of standard error.

A distinct trend was observed in the serine protease sensitivity assays with trypsin and chymotrypsin (Figure 14B). All peptides were highly susceptible to chymotrypsin, with only about 20% (peptides **2**, **6** and **7**) or 40% (peptide **12**) of the intact form remaining after 4 h. In contrast, all analogues displayed greater stability toward trypsin-mediated cleavage (>40% of intact peptide after 4 h), with the sole exception of peptide **7** which retained only 10% integrity after 4 h. Notably, peptide **12** demonstrated the highest resistance to trypsin, maintaining approximately 80 and 35% integrity after 4 and 24 h, respectively.

These results suggest that pendant guanidino substitutions at residues within the *N*-terminal segment exert a limited impact or may even enhance proteolytic susceptibility, depending on their spatial orientation, as evidenced by the contrasting behaviors of peptides **6** and **7**. Conversely, the superior stability of peptide **12** underscores the protective effect of the guanidino bridge, which likely promotes a more compact, partially helical conformation in solution, thereby reducing the accessibility of proteases to cleavage-prone residues.

## Conclusions

In this study, we implemented a guanidino-scanning strategy to improve the biological profile of cyclic temporin L-derived peptides. By strategically incorporating guanidino groups, either as pendant moieties or within the cyclizing bridge, we generated a series of analogues with enhanced antimicrobial activity, particularly against Gram-negative bacteria. Among them, peptides **7** and **12** emerged as the most promising candidates, showing up to 8-fold increased potency over the parent peptide **1**, while maintaining acceptable cytotoxicity toward human keratinocytes. A systematic SAR analysis revealed that biological improvements stemmed not merely from the increased cationic character, but from the specific spatial arrangement of guanidino groups within the sequence. Representative analogues were further investigated for hemolytic potential, antibiofilm effects, proteolytic stability, and mechanism of action, providing an integrated view of guanidino-driven effects on peptide behavior. Mechanistic studies confirmed that guanidino incorporation enhanced membrane interaction, promoted oligomerization within lipid bilayers, and facilitated membrane disruption, hallmarks of potent AMP activity. Notably, peptide **12** displayed strong insertion into model membranes, superior stability in human serum and toward proteolytic enzymes, and rapid bactericidal kinetics. Antibiofilm activity validated by fluorescence microscopy further highlighted the potential of peptides **7** and **12** to target sessile bacterial populations, which are a major clinical challenge in chronic infections. Collectively, these results validate guanidino-scanning as an effective strategy for fine-tuning the activity-selectivity balance of AMPs. This approach provides a versatile framework for the rational design of next-generation cyclic AMPs and lays a strong foundation for further structure-based optimization of guanidino-rich peptide scaffolds aimed at overcoming multidrug resistance.

## Experimental Section

### Materials and General Procedures

The *N* ^α^-Fmoc-protected amino acids, Arg­(Pbf), Glu­(*O*All), d-Glu­(*O*All), Ile, Leu, d-Leu, Lys­(Boc), Lys­(Alloc), Lys­(Mtt), d-Lys­(Mtt), Orn­(Alloc), Orn­(Mtt), Pro, Pro­(4-guanidino-Pbf)­(*2S*,*4S*), Phe, Phe­(*p*-guanidino-Boc_2_), Trp­(Boc), and Val were purchased from Iris-Biotech GmbH (Marktredwitz, Germany). Fmoc-β-Ala, Fmoc-GABA and Boc-Phe were purchased from Iris-Biotech GmbH (Marktredwitz, Germany). Activating and additive reagents such as (1-cyano-2-ethoxy-2-oxoethylidenaminooxy)­dimethylamino-morpholino-carbenium hexafluorophosphate (COMU), ethyl cyano­(hydroxyimino)­acetate (OxymaPure), 1-hydroxy-7-azabenzotriazole (HOAt), (7-azabenzotriazol-1-yloxy)­trispyrrolidinophosphonium hexafluorophosphate (PyAOP) were obtained commercially from GL Biochem Ltd. (Shanghai, China). The Fmoc-Leu-Rink Amide Resin (0.64 mmol/g of loading substitution) was purchased by Biosynth (Berkshire, United Kingdom). *N*,*N*-diisopropylethylamine (DIEA), piperidine, trifluoroacetic acid (TFA), triisopropylsilane (TIS) were obtained commercially from Merck Life Science (Milan, Italy). Mercury­(II) chloride (HgCl_2_), 1,3-bis­(*tert*-butoxycarbonyl)-2-methyl-2-thiopseudourea, di­(2-pyridyl)­thionocarbonate (DPT), iodomethane (CH_3_I), ammonium acetate (NH_4_OAc), *N*-methylmorpholine (NMM) and dimethyl sulfoxide (DMSO) were obtained commercially from Merck Life Science (Milan, Italy) or Iris-Biotech GmbH (Marktredwitz, Germany). Reagents for peptide cyclization, such as 1,3-dimethylbarbituric acid (NDMBA), tetrakis­(triphenylphosphine)­palladium(0) [Pd­(PPh_3_)_4_], sodium *N*,*N*-diethyldithiocarbamate, triethylamine (TEA) were purchased by Merck Life Science (Milan, Italy). Plastic syringe tubes (ISOLUTE SPE filtration column) equipped with Teflon filter (ISOLUTE frits, 20 μm porosity polyethylene frits) were acquired by Biotage, Uppsala, Sweden. The ultrasonic bath SONOREX RK 52 H was purchased by BANDELIN electronic (Berlin, Germany). Anhydrous solvents *N*,*N*-dimethylformamide (DMF), dichloromethane (DCM) and tetrahydrofuran (THF) were purchased by Merck Life Science (Milan, Italy). Solvents for peptide synthesis, such as *N*,*N*-dimethylformamide (DMF), dichloromethane (DCM), diethyl ether (Et_2_O), methanol (MeOH) and for HPLC analysis and purification, such as water and acetonitrile (MeCN), were of reagent grade acquired from commercial sources (Merck Life Science or VWR, Milan, Italy) and used without further purification.

Phospholipids: 1,2-dioleoyl-*sn*-glycero-3-phosphoethanolamine (DOPE), 1,2-dioleoyl-*sn*-glycero-3-phospho-(1′-rac-glycerol) sodium salt (DOPG), cardiolipin (CL) sodium salt (Heart, Bovine), Rhodamine-phosphatidylethanolamine (Rho-PE), 12-(*N*-methyl-*N*-(7-nitrobenz-2-oxa-1,3-diazol-4-yl)) (NBD)–phosphatidylethanolamine (NBD-PE), 1-palmitoyl-2–(4,5-dibromo)­stearoyl-*sn*-glycero-3-phosphocholine (4,5 Br-PC), and 1-palmitoyl-2–(11,12-dibromo)­stearoyl-*sn*-glycero-3- phosphocholine (11,12 Br-PC), were purchased from Avanti Polar Lipids (Birmingham, AL). 8-Aminonaphtalene-1,3,6-trisulfonic acid, disodium salt (ANTS), *p*-xylene-bis-pyridinium bromide (DPX), Laurdan, Thioflavin T (ThT), acrylamide, Triton, were purchased from Merck Life Science (Milan, Italy).

Purification of peptides was performed by RP-HPLC (Shimadzu Preparative Liquid Chromatograph LC-8A) equipped with a preparative column (Phenomenex Kinetex C18 column, 5 μm, 100 Å, 150 × 21.2 mm^2^) using linear gradients of MeCN (0.1% TFA) in water (0.1% TFA), from 10 to 70% over 20 min, with flow rate of 10 mL/min and UV detection at 220 nm. Final products were obtained by lyophilization of the appropriate fractions after removal of the MeCN by rotary evaporation. Analytical UHPLC (Shimadzu Nexera Liquid Chromatograph LC-30AD) analysis to evaluate the purity of peptides were performed on a Phenomenex Kinetex reversed-phase column (C18, 5 μm, 100 Å, 150 × 4.6 mm^2^) using a linear gradient of MeCN (0.1% TFA) in water (0.1% TFA), from 10 to 70% over 20 min, with flow rate of 1 mL/min and UV detection at 220 nm. All compounds examined for biological activity were purified to >97% (Figures S1–S12, Supporting Information), and the correct molecular ions were confirmed by ESI mass spectrometry (ESI-MS) (Figure S13, Supporting Information).

### Peptide Synthesis

Peptides were synthesized *via* stepwise Fmoc-based US-SPPS strategy.^29–31^ Assembly was carried out on Rink amide resin (0.25 mmol, 0.64 mmol/g loading, 100–200 mesh) placed in 10 mL plastic syringe reactors equipped with a frit and cap. The resin was swollen in DMF for 20 min at rt on an automated shaker. Fmoc deprotection was achieved using 20% piperidine in DMF (0.5 + 1 min), with the syringe reactor placed in an ultrasonic bath ensuring the reaction mixture remained below the water level. After each deprotection or coupling step, the resin was washed with DMF (2 mL × 3). Amino acid couplings were performed with 2 equiv of Fmoc-amino acid, COMU, and OxymaPure, and 4 equiv of DIEA in DMF under ultrasonic irradiation for 10 min. Reaction progress was monitored by Kaiser or Chloranil tests to detect resin-bound primary and secondary amines, respectively.

#### Allyl Deprotection and On-Resin Lactam Cyclization

Side-chain-to-side-chain lactam bridges of peptides **2–10** were performed on resin following known procedures.^22,33,34^ Allyl-based protecting groups on lysine and glutamic acid residues were removed by treating the resin, prewashed (with DCM, 2 mL × 3) and dried, with Pd­(PPh_3_)_4_ (0.15 equiv) and NDMBA (3 equiv) in dry DCM/DMF (3:2, v/v) under gentle shaking for 1 h. After filtration and washings with DMF (2 mL × 3) and DCM (2 mL × 3), the procedure was repeated. The resin was then treated with 0.5% sodium *N*,*N*-diethyldithiocarbamate in DMF (30 min), and complete removal was confirmed by LC-MS analysis of a cleaved resin aliquot [5 mg treated with 1 mL of TFA/TIS/H_2_O (95:2.5:2.5, v/v/v)]. Macrocyclization was performed using PyAOP (3 equiv), HOAt (3 equiv) and DIEA (6 equiv) in DMF/DCM (1:1 v/v) for 12 h at rt with shaking. The reaction was monitored by LC-MS, and the peptidyl resin was washed with DCM (2 mL × 3) and dried *under vacuum*.

#### On-Resin Guanylation

Peptides **3–5** contained *N*-terminal guanidino groups (replaced with the amino group of Phe, β-Ala and GABA) introduced *via* on-resin guanylation reaction.^34^ Following Fmoc deprotection, the resin-bound peptide was treated with HgCl_2_ (2 equiv), 1,3-bis­(*tert-*butoxycarbonyl)-2-methyl-2-thiopseudourea (2 equiv), and DIEA (2 equiv), suspended in DMF, and shaken for 16 h at rt. Afterward, the resin was sequentially filtered and washed with DMF (2 mL × 3), MeOH (2 mL × 3), and THF (2 mL × 3). Quantitative conversion to the guanidino group was confirmed by LC-MS analysis of a cleaved resin aliquot.

#### Guanidino Bridge Formation

For peptides **11–13**, guanidino bridges were formed between two diaminoacyl residues protected with Alloc and Mtt groups, respectively.^26,32^ Following allyl deprotection as described above, the exposed side-chain amine was converted to isothiocyanate by treatment with DPT (5 equiv) in DCM under shaking for 12 h. After LC-MS confirmation, the Mtt group of the second diaminoacyl residue was removed using a cocktail of DCM/TIS/TFA (94:5:1, v/v/v) with repeated 20 min treatments (3 mL × 10), followed by washing with DMF, MeOH, and DCM. Mtt removal was ascertained by colorimetric Kaiser test. The thiourea linkage was formed by suspending the resin in anhydrous THF with TEA (10 equiv) and shaking the mixture for 12 h at rt. After LC-MS confirmation, *S*-methylation was performed by treating the resin with a 0.2 M solution of CH_3_I in DMF (15 mL) for 3 × 1 h. The final guanidino bridge was formed by treating the resin with 2 M NH_4_OAc solution in DMSO (3 mL) and NMM (1 equiv), incubated at 80 °C for 12 h. The resin was washed with DMF (2 mL × 3) and DCM (2 mL × 3). Completion of this step was monitored by LC-MS, and the resin was washed and dried as above.

#### Peptide Cleavage

Following complete peptide chain assembly cyclization, all resin-bound peptides were cleaved from the resin using TFA/TIS/H_2_O (95:2.5:2.5, v/v/v) for 3 h at rt. The filtrates were precipitated with chilled Et_2_O, centrifugated (15 min, 600 rpm), and the resulting crude peptides were dried under *vacuum* and dissolved in 10% MeCN in H_2_O for purification by RP-HPLC.

### Antimicrobial Activity

Bacterial strains used in this study were the Gram-positive *S. aureus* ATCC 25923, *S. epidermidis* ATCC 12228, *B. megaterium* BM11, *S. agalactiae* ATCC 27591, *E. faecalis* ATCC 29212 and the Gram-negative *E. coli* ATCC 25922, *P. aeruginosa* ATCC 27853, *A. baumannii* ATCC 19606, *E. coli* D21 and *P. aeruginosa* PAO1. The minimal inhibitory concentrations (MIC) were defined as the lowest concentration of the peptide that completely inhibited bacterial growth, after incubation for 16–18 h at 37 °C and were determined by the broth microdilution assay as previously described.^47^ Bacterial cells in the mid log phase were diluted in Muller Hinton (MH) at a concentration of 2 × 10^6^ cell/mL. Aliquots of 50 μL of this bacterial suspension were added to 50 μL of MH containing serial 2-fold dilutions of the peptides (from 100 to 0.19 μM) in the wells a 96-well plate. Ciprofloxacin was used as antibiotic control. The plate was then incubated at 37 °C. MICs were obtained from three identical readings of four independent experiments.

### Cytotoxicity and Hemolytic Assay

For the evaluation of peptides’ cytotoxicity, the human immortalized keratinocytes (HaCaT cells), purchased from AddexBio (San Diego, CA, USA), and BEAS-2B were used. HaCaT cells were cultured in Dulbecco’s modified Eagle’s medium supplemented with 4 mM glutamine (DMEM), 10% heat-inactivated fetal bovine serum (FBS), and 0.1 mg/mL of penicillin and streptomycin, at 37 °C and 5% CO_2_. BEAS-2B cells were cultured in RPMI, supplemented with 2 mM glutamine (DMEM), 10% heat-inactivated fetal bovine serum (FBS), 1 mM sodium pyruvate, and 0.1 mg/mL of penicillin and streptomycin, at 37 °C and 5% CO_2_. Cytotoxicity was tested *in vitro* using a colorimetric assay that relies on the intracellular reduction of the yellow 3-(4,5-dimethylthiazol-2-yl)-2,5-diphenyltetrazolium bromide salt (MTT) by mitochondrial dehydrogenases of metabolically active cells in formazan crystals (purple). About 4 × 10^4^ HaCaT and BEAS-2B cells, suspended in DMEM supplemented with 2% FBS, were plated in triplicate wells of a 96-well microtiter plate. The plate was incubated overnight at 37 °C and 5% CO_2_. Then, the cells were treated with different concentrations of the peptide (from 100 to 3.12 μM) in a fresh serum-free medium for 2 h and for a longer time (24 h). Following peptide treatment, the medium was removed, and MTT (0.5 mg/mL) solution in Hank’s buffer (136 mM NaCl, 4.2 mM Na_2_HPO_4_, 4.4 mM KH_2_PO_4_, 5.4 mM KCl, 4.1 mM NaHCO_3_, pH 7.2, supplemented with 20 mM d-glucose) was added to each well of the plate, that was then incubated for 4 h at 37 °C and 5% CO_2_. The purple formazan crystals were dissolved using acidified 2-propanol and a microplate reader (Infinite M200; Tecan, Salzburg, Austria) was used to measure the absorbance of each well at 570 nm. The percentage of cell viability was calculated compared to the control, which consisted of cells without any peptide treatment (corresponding to 100% viability) and all data represent the mean of three independent experiments ± SEM.

The hemolytic activity of peptides **2**, **6**, **7** and **12** analogs was assessed by quantifying the release of hemoglobin resulting from the disruption of defibrinated sheep RBCs (OXOID, SR0051D, Milan, Italy). Erythrocytes were washed twice in 0.9% (w/v) NaCl and the suspension was adjusted to an OD of 0.5 at 500 nm in 0.9% (w/v) NaCl. RBCs were then incubated with peptide solutions at concentrations ranging from 1.56 to 50 μM or vehicle (water, as negative control) for 30 min at 37 °C with gentle agitation. After incubation, the samples were centrifuged at 900*g* for 5 min, and hemoglobin released in the supernatant was quantified by measuring absorbance at 415 nm using a microplate reader (Infinite M200; Tecan, Salzburg, Austria). Complete lysis of erythrocytes (100%) was obtained using distilled water. The data were reported as the mean ± the standard deviation (SD) of three independent experiments.

### Antibiofilm Activity and Fluorescence Microscopy

The antibiofilm activity was evaluated as elsewhere reported.^48^ Bacteria grew at 37 °C until they reached an optical density (OD) of 0.8 (λ = 590 nm). The culture was diluted to a cell density of 1 × 10^6^ colony-forming units (CFUs)/mL and aliquots of 100 μL of this suspension were dispensed into the wells of a 96-multiwell plate. After biofilm formation (incubation time = 20 h at 37 °C), all the planktonic cells were removed, and the wells were washed twice with PBS (150 μL). After washing, the wells were filled with PBS supplemented with different 2-fold serial dilutions of the tested peptides (from 50 to 1.56 μM). After peptide treatment (incubation time = 2 h at 37 °C) the wells were washed with PBS twice and filled with 150 μL of MTT (0.5 mg/mL) to evaluate biofilm viability after 4 h incubation at 37 °C. The reaction was then stopped by adding SDS (final concentration equal to 5% v/v). A microplate reader (Infinite M200; Tecan, Salzburg, Austria) was used to measure the absorbance of each well at 570 nm and the percentage of biofilm viability was calculated relative to the untreated samples.

For fluorescence microscopy analysis, biofilms of both *S. aureus* ATCC 25923 and *P. aeruginosa* ATCC 27853 were formed under the same conditions as described above, but on coverslips immersed in microbial culture. Afterward, the coverslips were rinsed twice with sterile 0.85% NaCl and treated for 2 h at different concentrations of **7** and **12** (*i*.*e*., 6.25 and 25 μM against *S. aureus* and 12.5 and 50 μM against *P. aeruginosa*, according to their antibiofilm activity). After treatment, the coverslips were stained with SYTO 9 and PI at concentrations 6 and 30 μM, respectively, as elsewhere reported.^49^ After 15 min of incubation at room temperature in the dark, the coverslips were rinsed twice with 0.85% NaCl and placed on a glass slide for the examination under a fluorescence microscope. Fluorescence images were recorded using an Olympus optical microscope (×40).

### Trp Quenching in Solution and LUVs

The peptide insertion in LUVs was monitored measuring the incorporation of Tryptophan located in position 4 inside the lipid bilayer. LUVs mimicking Gram-positive and Gram-negative membranes, were prepared as described above. To monitor the Trp insertion inside LUVs, the Trp residue was quenched with the quencher acrylamide that is soluble in water and is not able to partition in liposomes. The experiment was performed in solution and LUVs with different compositions. The peptide was dissolved in buffer at the concentration of 10 μM and Trp was quenched with the increasing acrylamide concentrations from 0.02 to 0.22 M. Each Trp spectrum was recorded exciting at 295 nm and fluorescence emission was measured at 355 nm for the calculation of the Stern–Volmer equation according to the following equation: *F*_0_/*F* = 1 + *K*_sv_ [Q], where *F*_0_ and *F* represent the fluorescence intensities in the absence and the presence of the quencher (*Q*), respectively.^48^ The same experiment was performed in LUVs (250 μM) where the peptide (10 μM) was incubated with the liposomes for 10 min and then the Trp residue was quenched with acrylamide concentrations from 0.02 M to 0.22M. Each Trp spectrum was recorded exciting at 295 nm and fluorescence emission was measured at 355 nm for the calculation of the Stern–Volmer equation as described above.

Moreover, we also studied Trp insertion using LUVs containing phospholipids labeled with bromo probe that is considered a Trp quencher as well as acrylamide.^50^ We prepared LUVs containing 25% of phospholipids with bromo in different positions; specifically, we used 4,5 Br-PC and 11,12 Br-PC. Lipid films were prepared at a final concentration of 250 μM and then were treated to obtain LUVs. LUVs were incubated with 10 μM of peptides for 5 min and each Trp spectrum emission was recorded exciting at 295 nm.

### Peptide Aggregation

The peptide aggregation in the presence of LUVs mimicking Gram-positive, Gram-negative, and eukaryotic membranes, was studied using ThT as fluorescent dye. Upon binding to peptide aggregates, when ThT is excited at 450 nm it gives an increase of its fluorescence signal at 482 nm.^51^

The ThT experiment was performed preparing LUVs with different phospholipid compositions to mimic a specific membrane environment. Specifically, we used: (i) LUVs made of DOPG:CL (58:42, mol/mol) to mimic Gram-positive membrane; (ii) LUVs made of DOPE:DOPG:CL (65:23:12, mol/mol/mol) to mimic Gram-negative membrane. LUVs were obtained using extrusion method as previously described.^22^ First, we prepared the lipid film at the concentration of 100 μM dissolving the phospholipids in chloroform and taking the appropriate amount in according to the LUVs’ composition. After the assembly of phospholipids, the organic solvent was removed, and the lipid film was obtained after the lyophilization. Then, the lipid film was hydrated with 100 mM NaCl, 10 mM Tris–HCl, 25 μM Tht buffer pH 7.4 and vortexed for 1 h; then the lipid suspension was freeze–thawed 6 times and extruded 10 times through polycarbonate membranes with 0.1 μm diameter pores to obtain LUVs.^48^

To evaluate the aggregation, LUVs were titrated with the increasing peptide concentrations ranging from 5 to 50 μM and each ThT spectrum was recorded excited at 450 nm (slit width, 10 nm) and measuring fluorescence emission at 482 nm. Aggregation was calculated according to the following equation: %*A* = (*F*_f_ – *F*_0_)/(*F*_max_ – *F*_0_) × 100, where *F*_f_ indicates the fluorescence after peptide addition, *F*_0_ the initial fluorescence in the absence of peptide and *F*_max_ is the fluorescence maximum obtained immediately after peptide addition.

### Laurdan Assay

The change of membrane fluidity was investigated using Laurdan as fluorescent dye.^52^ LUVs with different compositions were prepared at the concentrations of 100 μM and Laurdan was encapsulated in the lipid film at the concentration of 1 μM. After the lyophilization, the lipid film was hydrated with PBS 1× buffer, pH 7.4, and treated using the extrusion method to obtain LUVs. To evaluate the change in membrane fluidity, LUVs were treated with each peptide at the concentrations of 25 μM and 50 μM, and Laurdan emission was recorded in the range from 400 to 550 nm with λ_ex_ 365 nm, where its emission can shift from an ordered phase at 440 nm to disordered phase at 490 nm. The effect on membrane fluidity before and after the peptide treatment was evaluated by calculating Generalized Polarization (GP) parameter as follows: GP = (*I*_440_ – *I*_490_)/(*I*_440_ + *I*_490_), where *I*_440_ and *I*_490_ indicate the fluorescence intensities at the maximum emission wavelength in the ordered and disordered, respectively.^22^

### Circular Dichroism (CD)

The secondary structure of each peptide was studied in solution and in small unilamellar vesicles (SUVs) mimicking Gram-positive and Gram-negative membranes performing CD analysis. The CD spectrum for each peptide in solution was recorded at 150 μM, using the quartz cells of 0.1 cm path length with a Jasco J-1500 CD Spectrometer at 25 °C. SUVs at the concentration of 150 μM (*P*/*L* = 1) and 50 μM (*P*/*L* = 0.4) were prepared as reported: the lipid film was hydrated with water for 1h and then sonicated for 30 min. CD spectra were recorded at the peptide/lipid ratio of 1:1 both in the presence with SUVs mimicking Gram-positive and Gram-negative membranes. CD spectra were recorded at 25 °C on a in a 0.1 cm quartz cell using three consecutive scans from 260 to 190 nm, 3 nm bandwidth, a time constant of 16 s, and a scan rate of 10 nm/min.

### Fusion Assay

The fusogenic ability of peptides was studied performing the lipid mixing experiment and evaluating a reduction in the fluorescence energy transfer efficiency between fluorophores 12-(*N*-methyl-*N*-(7-nitrobenz-2-oxa-1,3-diazol-4-yl)) (NBD) and Rhodamine (Rho) bound to liposomes.^42^ We prepared unlabeled LUVs mimicking Gram-positive and Gram-negative membranes at a concentration of 0.16 mM, and labeled LUVs mimicking Gram-positive and Gram-negative membranes with 0.6% mol of NBD-PE and 0.6% mol of Rho-PE at the concentration of 0.04 mM. LUVs were prepared as described above, and for the experiment, unlabeled and labeled LUVs were mixed at a 1:4 ratio to have final lipid concentration of 0.1 mM.^53^ The fusogenic activity of each peptide was evaluated at lipide/peptide ratio of 0.01, 0.03, 0.05, 0.1, 0.2, 0.3, 0.5, and NBD emission at 530 nm and Rho emission at 590 nm were recorded setting an NBD excitation wavelength set at 465 nm.

The percentage of fusion was calculated as a function of 100% value corresponding to complete mixing of LUVs upon the addition of Triton X-100 (0.05% v/v). The percentage fusion was calculated as

$$\frac{\left[F_{530}/F_{590}\right]\mathrm{peptide}-\left[F_{530}/F_{590}\right]\mathrm{blank}}{\left[F_{530}/F_{590}\right]\mathrm{triton}-\left[F_{530}/F_{590}\right]\mathrm{blank}}\times 100$$

where *F*_530_ and *F*_590_ are the fluorescence intensities at 530 and 590 nm calculated in the absence and in the presence of the peptide and Triton X-100.

### Leakage Assay

The peptide ability to induce the membrane damage causing the pore formation was evaluated by performing the leakage assay using ANTs and DPX as probes.^22^ We prepared LUVs mimicking Gram-positive and Gram-negative membranes encapsulating ANTs and DPX. Once the lipid films were obtained as described above, ANTs (12.5 mM) and DPX (45 mM) dissolved in water were added, and the mixture was lyophilized overnight. Then, the lipid films were hydrated with PBS 1× buffer for 1h and extruded for obtaining LUVs. The excess of both fluorophores was removed by performing gel filtration using a Sephadex G-50 column (1.5 cm × 10 cm). To evaluate liposome leakage by measuring the dequenching of ANTs from DPX that are kept together by collisional transfer,^54^ LUVs were treated with increasing peptide concentrations from 5 to 250 μM. ANTs fluorescence released in the medium was recorded at 512 nm setting an excitation fluorescence at 385 nm before and after the peptide addition. The percentage of LUVs’ leakage was calculated as %leakage = (*F*_i_ – *F*_0_)/(*F*_t_– *F*_0_), where *F*_0_ indicates the fluorescence of intact LUVs before the peptide addition, while *F*_i_ and *F*_t_ are fluorescence intensities after peptide and Triton-X (0.1%) treatment, respectively.

### Membrane Permeabilization Assay and Killing Kinetics

The ability of peptides to alter the bacterial membrane permeability of planktonic cells of *S. aureus* ATCC 25923 and *P. aeruginosa* ATCC 27853 was determined by the Sytox Green assay. Approximately 1 × 10^6^ cells in 100 μL of PBS were combined with 1 μM Sytox Green in the dark for 5 min. The fluorescence intensity was monitored using a microplate reader (Infinite M200, Tecan), with excitation and emission at λ = 485 and 535 nm, respectively. Peptides were added (time = 0) at three different concentrations (*i*.*e*., 3.12, 6.25, and 12.5 μM). Increase in the fluorescence intensity is due to the binding of the dye to intracellular nucleic acids. Controls consisted of cells that were not exposed to the peptides. The values correspond to one representative experiment out of three independent experiments.

For the killing kinetics, 10^6^ CFU/mL were incubated with peptide **7** and **12** at three different concentrations corresponding to 2 × MIC, MIC, and 1/2 × MIC. Aliquots were withdrawn after 5, 30, and 120 min, appropriately diluted in PBS, and spread onto agar plates for CFU counting after overnight incubation. Controls were samples in the presence of the peptide solvent.

### Proteolytic Stability Assays

Peptide stability was evaluated by modifying previously described procedures.^45,46^ The assays were performed in human serum and in the presence of proteolytic enzymes such as trypsin and chymotrypsin. For human serum stability, 2 mM peptide stock solutions were prepared in water and mixed with human serum (from male AB plasma, USA origin, sterile-filtered, Merck/Sigma-Aldrich, USA) to obtain a final concentration of 200 μM [human plasma/stock solution (9:1, v/v)]. The mixtures were incubated at 37 ± 1 °C, and 50 μL aliquots were taken at predetermined time intervals (0, 1, 2, 4, 8, and 24 h). Protease activity was quenched by adding 100 μL of acetonitrile (MeCN). The resulting cloudy suspensions were cooled to 4 °C for 30 min and centrifugated at 12,000 rpm for 10 min to remove the serum proteins. Then, the supernatants were analyzed by analytical HPLC (Shimadzu Nexera Liquid Chromatograph LC-30AD) which was equipped with a Phenomenex Kinetex reversed-phase column (C18, 5 μm, 100 Å, 150 mm × 4.6 mm) with a flow rate of 1 mL/min using a gradient of MeCN (0.1% TFA) in water (0.1% TFA), from 10 to 70% over 15 min, and UV detection at 220 nm. For enzyme stability, each peptide peptide (200 μM) was incubated with trypsin (0.5 μM) in PBS 1× (pH 7.2) or with chymotrypsin (0.005 μM) in Tris–HCl (100 mM, pH 7.8). Samples (50 μL) were collected at 0, 1, 2, 4, and 24 h, quenched with 0.5% TFA in water, and analyzed by HPLC under chromatographic conditions as described above. All experiments were conducted in triplicate. The percentage of intact peptides at each time point was determined by integrating the corresponding HPLC peak areas.

## Supplementary Material





## Notes

Analytical data of peptides **2–13** (Table S1); HPLC traces for the synthesized peptides (Figures S1–S12); MS spectra of the synthesized peptides (Figure S13); CD spectra in water for the synthesized peptides (Figure S14); cytotoxicity against HaCaT and BEAS-2B cells after 2 and 24 h exposure (Tables S2 and S3); generalized polarization (GP) values calculated in membrane fluidity assay (Table S4); fluorescence images of Sytox Green-stained cells showing effects of peptide **12** on membrane permeability (Figure S15) (PDF)
Molecular formula strings (CSV)

## Abbreviations Used

ANTS: 8-aminonaphthalene-1,3,6-trisulfonic acid, disodium salt
CC_50_: 50% cytotoxic concentration
CL: cardiolipin
COMU: (1-cyano-2-ethoxy-2-oxoethylidenaminooxy)­dimethylamino-morpholino-carbenium hexafluorophosphate
DMEM: Dulbecco’s modified eagle medium
DOPE: 1,2-dioleoyl-*sn*-glycero-3-phosphoethanolamine
DOPG: 1,2-dioleoyl-*sn*-glycero-3-phosphoglycerol
DPC: dodecylphosphorylcholine
DPT: di­(2-pyridyl)­thionocarbonate
DPX: *p*-xylene-bis-pyridinium bromide
GP: generalized polarization
HOAt: 1-hydroxy-7-azabenzotriazole
LDLR: low-density lipoprotein receptor
LUV: large unilamellar vesicle
MIC: minimal inhibitory concentrations
Mtt: 4-methyltrityl
MTT: 3-(4,5-dimethylthiazol-2-yl)-2,5-diphenyltetrazolium bromide
NBD: nitrobenzoxadiazoles
NDMBA: 1,3-dimethylbarbituric acid
NMM: *N*-methylmorpholine
Orn: Ornitine
Oxyma: ethyl cyano­(hydroxyimino)­acetate
Pd­(PPh_3_)_4_: tetrakis­(triphenylphosphine)­palladium­(0)
PyAOP: (7-azabenzotriazol-1-yloxy)­trispyrrolidinophosphonium hexafluorophosphate
Rho: rhodamine
TEA: triethylamine
ThT: Thioflavin T
TIS: triisopropylsilane
TL: temporin L
US-SPPS: ultrasonic-assisted solid-phase peptide synthesis
