# Supplementary material for: Probing Guanidino Pendant or Bridged Groups in Cyclic Antimicrobial Peptides Derived from Temporin L: A Strategy to Improve Efficacy against Gram-Negative Bacteria
Source: J Med Chem. 2025 Dec 1;68(23):25038–60. doi: 10.1021/acs.jmedchem.5c01984 (PMC12703733; doi:10.1021/acs.jmedchem.5c01984)
Supplement: Supplementary file 1 [file jm5c01984_si_001.pdf]

Supporting information

Probing Guanidino Pendant or Bridged Groups in Cyclic Antimicrobial Peptides Derived from Temporin L: a Strategy to Improve Efficacy against Gram-negative Bacteria

Rosa Bellavita,[a,#] Ida Boccino,[a,#] Maria Rosa Loffredo,[b,#] Sara Palladino,[a] Floriana Cappiello,[b] Carlo Vetrano,[b] Eeva Tortellini,[b] Vincenzo Mazzarella,[c] Salvatore Di Maro,[c] Stefania Galdiero,[a] Bruno Casciaro,[b,*] Paolo Grieco,[a] Maria Luisa Mangoni[b,§] and Francesco Merlino[a,§,*]

[a]*Department of Pharmacy, University of Naples Federico II, via Domenico Montesano 49, 80131, Naples, Italy*
[b]*Department of Biochemical Sciences, Laboratory Affiliated to Istituto Pasteur Italia-Fondazione Cenci Bolognetti, Sapienza University of Rome, p.le Aldo Moro 5, Rome 00185, Italy*
[c]*DiSTABiF, University of Campania Luigi Vanvitelli, via Vivaldi 43, 81100 Caserta, Italy*

[*]Email: bruno.casciaro@uniroma1.it; francesco.merlino@unina.it

Table of contents

1. Analytical data of peptides **2-13** (Table S1)	S2
2. HPLC chromatograms of peptides **2-13** (Figures S1-12)	S3
3. MS spectra of peptides **2-13** (Figure S13)	S9
4. CD spectra in water for peptides **2-13** (Figure S14)	S11
5. Cytotoxicity against HaCaT cells after 2 and 24 h exposure (Table S2)	S12
6. Cytotoxicity against BEAS-2B cells after 2 and 24 h exposure (Table S3)	S13
7. Generalized polarization (GP) values calculated in membrane fluidity assay (Table S4)	S14
8. Fluorescence images of Sytox Green-stained cells (Figure S15)	S16

Table S1. Analytical data of guanidino-based cyclic TL-derived peptides (**2-13**).

Peptide ID	t_R (min)	Molecular Formula	$[M+H]^+_{calcd}$	$[(M+nH)/n]^+_{obs}$
**2**	14.0	$C_{89}H_{131}N_{20}O_{14}^+$	1705.2	1705.0, n=1<br>852.8, n=2<br>569.1, n=3
**3**	14.3	$C_{90}H_{133}N_{22}O_{14}^+$	1747.2	1747.0, n=1<br>873.9, n=2<br>583.3, n=3
**4**	14.3	$C_{93}H_{138}N_{23}O_{15}^+$	1818.2	1818.0, n=1<br>909.3, n=2<br>606.9, n=3
**5**	13.2	$C_{94}H_{140}N_{23}O_{15}^+$	1832.3	1832.1, n=1<br>916.4, n=2<br>611.6, n=3
**6**	13.1	$C_{90}H_{134}N_{23}O_{14}^+$	1762.2	1762.0, n=1<br>881.4, n=2<br>588.2, n=3
**7**	12.1	$C_{90}H_{134}N_{23}O_{14}^+$	1762.2	1762.0, n=1<br>881.3, n=2<br>588.2, n=3
**8**	12.8	$C_{90}H_{134}N_{23}O_{14}^+$	1762.2	1762.0, n=1<br>881.4, n=2<br>588.2, n=3
**9**	14.1	$C_{89}H_{131}N_{22}O_{14}^+$	1733.2	1732.9, n=1<br>866.9, n=2<br>578.5, n=3
**10**	12.0	$C_{90}H_{134}N_{23}O_{14}^+$	1762.2	1762.1, n=1<br>881.6, n=2<br>588.3, n=3
**11**	14.5	$C_{91}H_{137}N_{22}O_{13}^+$	1747.2	1747.0, n=1<br>873.9, n=2<br>583.2, n=3
**12**	12.8	$C_{91}H_{137}N_{22}O_{13}^+$	1747.2	1747.0, n=1<br>873.9, n=2<br>583.2, n=3
**13**	14.4	$C_{89}H_{133}N_{22}O_{13}^+$	1719.2	1719.0, n=1<br>859.9, n=2<br>573.7, n=3

*HPLC chromatograms of peptides **2-13**.*

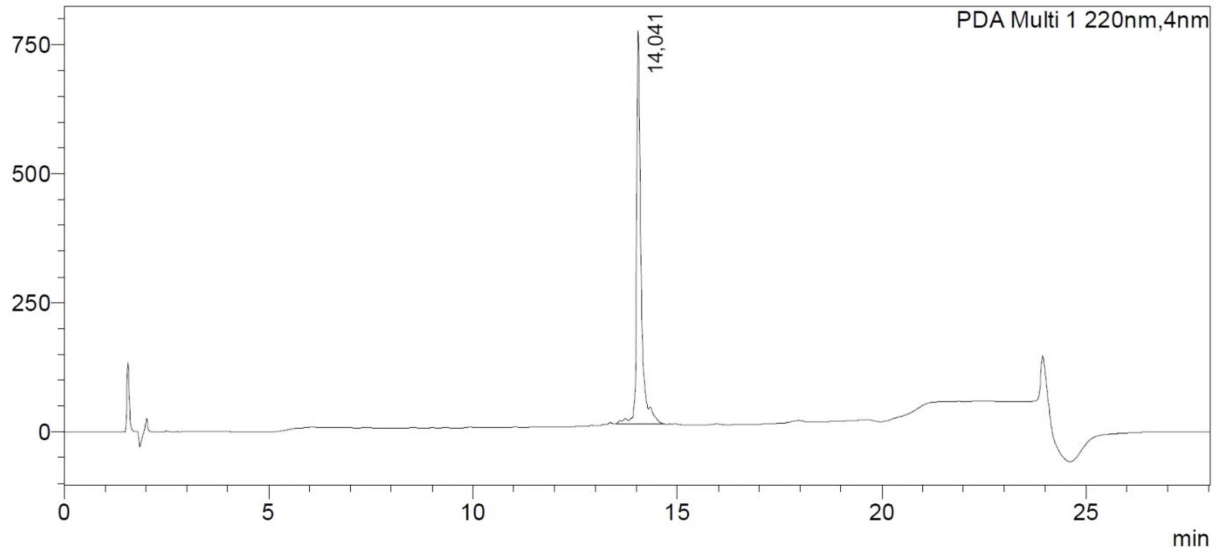

Figure S1. Analytical HPLC chromatogram of **2**, recorded using Shimadzu Nexera Liquid Chromatograph (SPD-M20A/DGU-20A3R/LC-20AD) with a Phenomenex Kinetex C18 column (150 × 4.6 mm, 5 µm, 100 Å). UV detection was performed at 220 nm. Retention time (t_R): 14.0 min [elution: linear gradient 10-70% MeCN (0.1% TFA) in H_2O (0.1% TFA) over 15 min; flow rate: 1 mL/min].

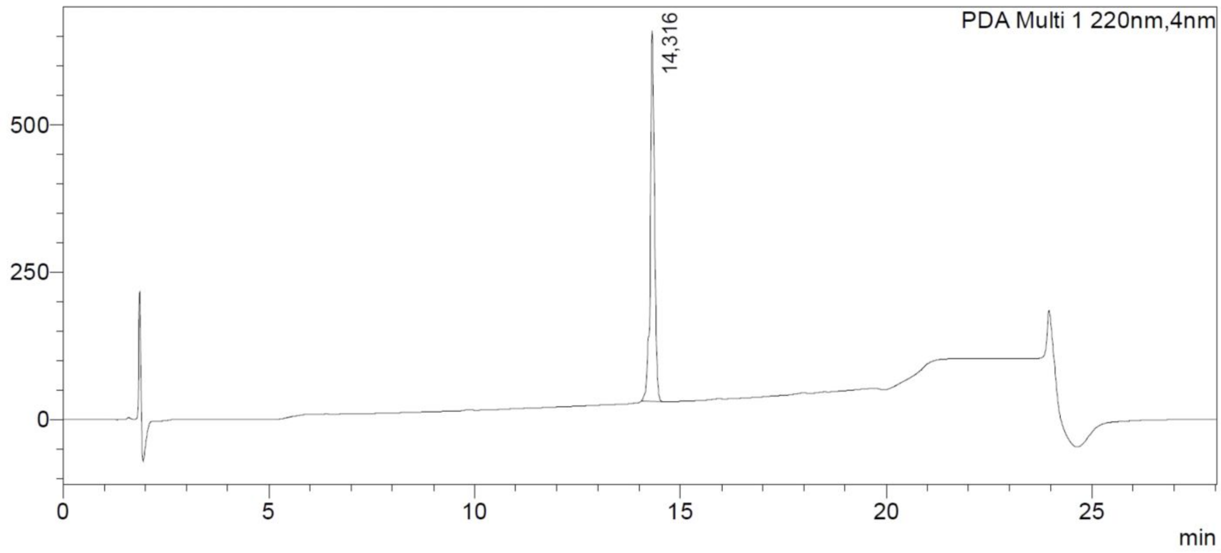

Figure S2. Analytical HPLC chromatogram of **3**, recorded using Shimadzu Nexera Liquid Chromatograph (SPD-M20A/DGU-20A3R/LC-20AD) with a Phenomenex Kinetex C18 column (150 × 4.6 mm, 5 µm, 100 Å). UV detection was performed at 220 nm. Retention time (t_R): 14.3 min [elution: linear gradient 10-70% MeCN (0.1% TFA) in H_2O (0.1% TFA) over 15 min; flow rate: 1 mL/min].

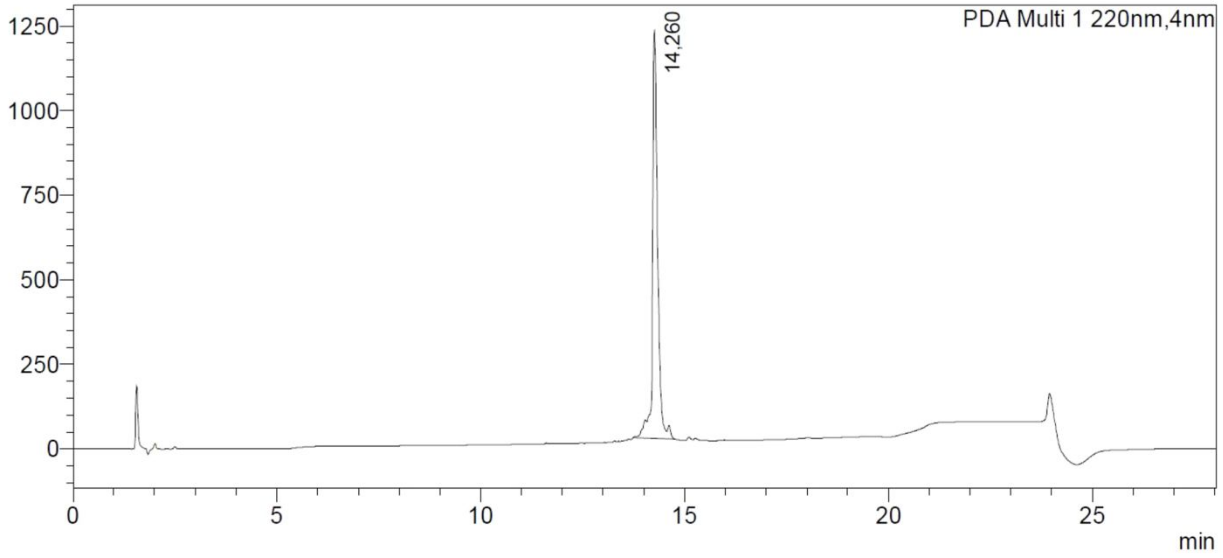

Figure S3. Analytical HPLC chromatogram of **4**, recorded using Shimadzu Nexera Liquid Chromatograph (SPD-M20A/DGU-20A3R/LC-20AD) with a Phenomenex Kinetex C18 column (150 × 4.6 mm, 5 µm, 100 Å). UV detection was performed at 220 nm. Retention time (t_R): 14.3 min [elution: linear gradient 10-70% MeCN (0.1% TFA) in H_2O (0.1% TFA) over 15 min; flow rate: 1 mL/min].

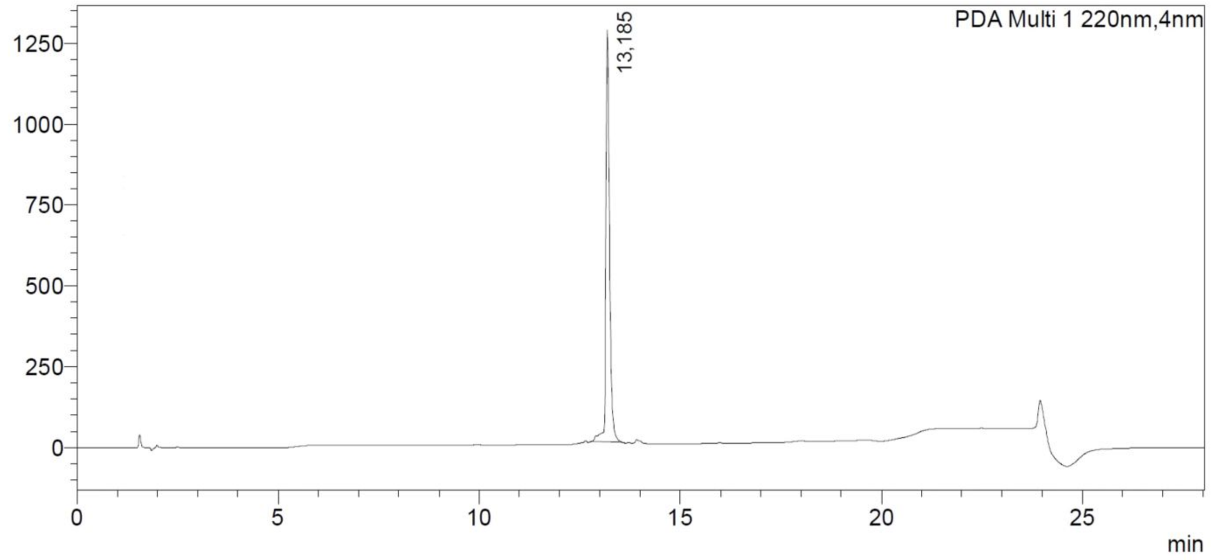

Figure S4. Analytical HPLC chromatogram of **5**, recorded using Shimadzu Nexera Liquid Chromatograph (SPD-M20A/DGU-20A3R/LC-20AD) with a Phenomenex Kinetex C18 column (150 × 4.6 mm, 5 µm, 100 Å). UV detection was performed at 220 nm. Retention time (t_R): 13.2 min [elution: linear gradient 10-70% MeCN (0.1% TFA) in H_2O (0.1% TFA) over 15 min; flow rate: 1 mL/min].

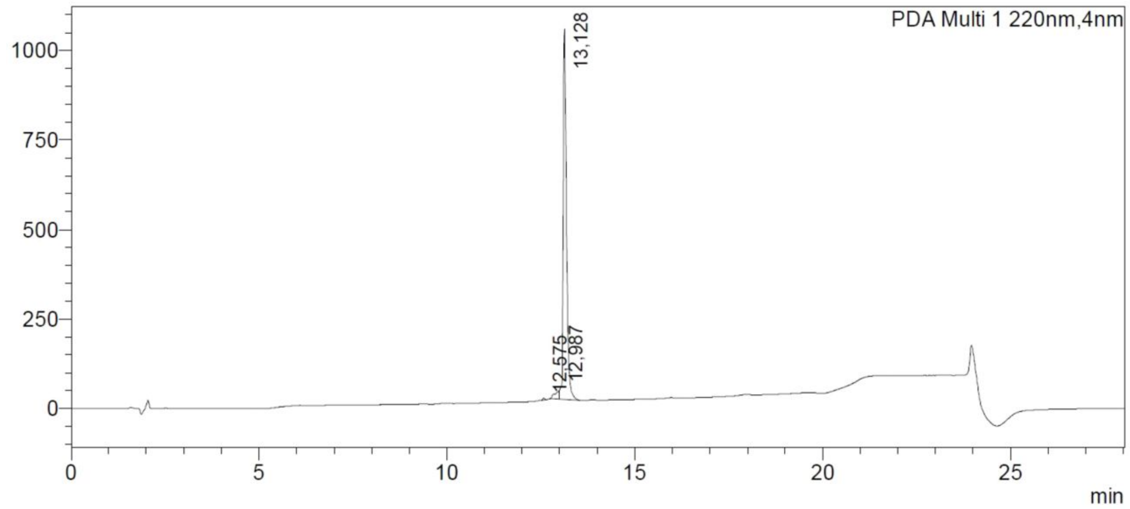

PDA Ch1 220nm

Peak#	Ret. Time	Area	Height	Area%
1	12,575	18768	5037	0,293
2	12,987	194642	22663	3,040
3	13,128	6188392	1034618	96,666
Total		6401802	1062318	100,000

Figure S5. Analytical HPLC chromatogram of **6**, recorded using Shimadzu Nexera Liquid Chromatograph (SPD-M20A/DGU-20A3R/LC-20AD) with a Phenomenex Kinetex C18 column (150 × 4.6 mm, 5 µm, 100 Å). UV detection was performed at 220 nm. Retention time (t_R): 13.1 min [elution: linear gradient 10-70% MeCN (0.1% TFA) in H_2O (0.1% TFA) over 15 min; flow rate: 1 mL/min]. Purity >96%.

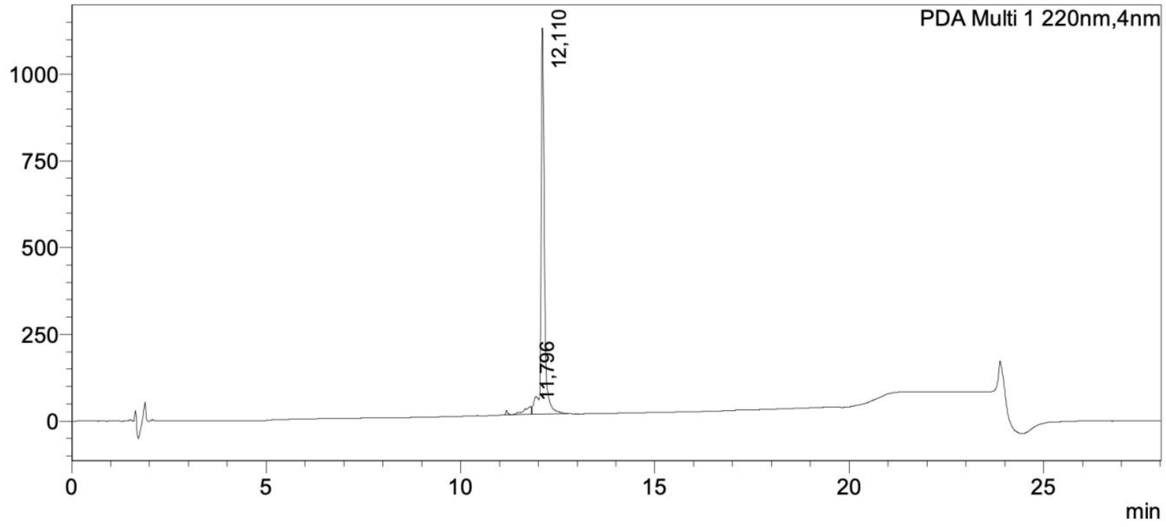

PDA Ch1 220nm

Peak#	Ret. Time	Area	Height	Area%
1	11,796	320547	21494	4,503
2	12,110	6798342	1112828	95,497
Total		7118889	1134322	100,000

Figure S6. Analytical HPLC chromatogram of **7**, recorded using Shimadzu Nexera Liquid Chromatograph (SPD-M20A/DGU-20A3R/LC-20AD) with a Phenomenex Kinetex C18 column (150 × 4.6 mm, 5 µm, 100 Å). UV detection was performed at 220 nm. Retention time (t_R): 12.1 min [elution: linear gradient 10-70% MeCN (0.1% TFA) in H_2O (0.1% TFA) over 15 min; flow rate: 1 mL/min]. Purity >95%.

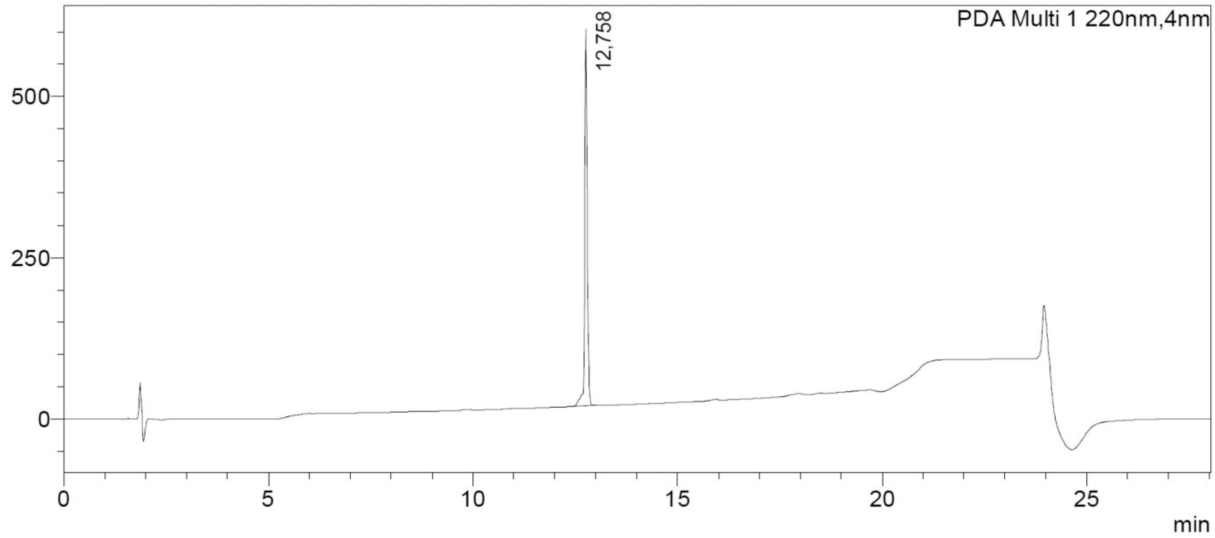

Figure S7. Analytical HPLC chromatogram of **8**, recorded using Shimadzu Nexera Liquid Chromatograph (SPD-M20A/DGU-20A3R/LC-20AD) with a Phenomenex Kinetex C18 column (150 × 4.6 mm, 5 µm, 100 Å). UV detection was performed at 220 nm. Retention time (t_R): 12.8 min [elution: linear gradient 10-70% MeCN (0.1% TFA) in H_2O (0.1% TFA) over 15 min; flow rate: 1 mL/min].

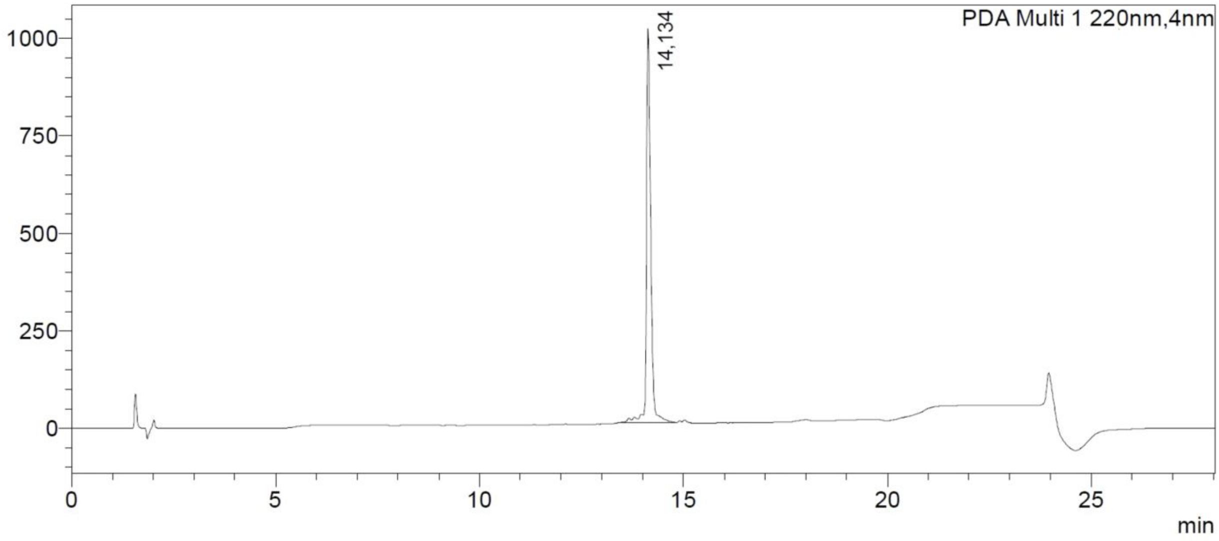

Figure S8. Analytical HPLC chromatogram of **9**, recorded using Shimadzu Nexera Liquid Chromatograph (SPD-M20A/DGU-20A3R/LC-20AD) with a Phenomenex Kinetex C18 column (150 × 4.6 mm, 5 µm, 100 Å). UV detection was performed at 220 nm. Retention time (t_R): 14.1 min [elution: linear gradient 10-70% MeCN (0.1% TFA) in H_2O (0.1% TFA) over 15 min; flow rate: 1 mL/min].

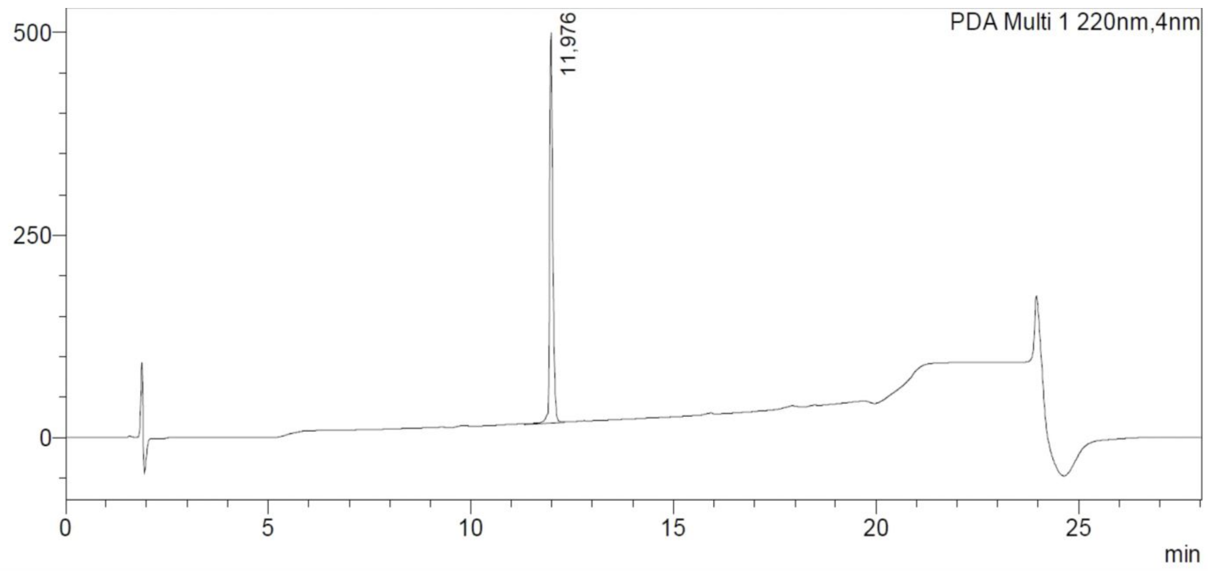

Figure S9. Analytical HPLC chromatogram of **10**, recorded using Shimadzu Nexera Liquid Chromatograph (SPD-M20A/DGU-20A3R/LC-20AD) with a Phenomenex Kinetex C18 column (150 × 4.6 mm, 5 µm, 100 Å). UV detection was performed at 220 nm. Retention time (t_R): 12.0 min [elution: linear gradient 10-70% MeCN (0.1% TFA) in H_2O (0.1% TFA) over 15 min; flow rate: 1 mL/min].

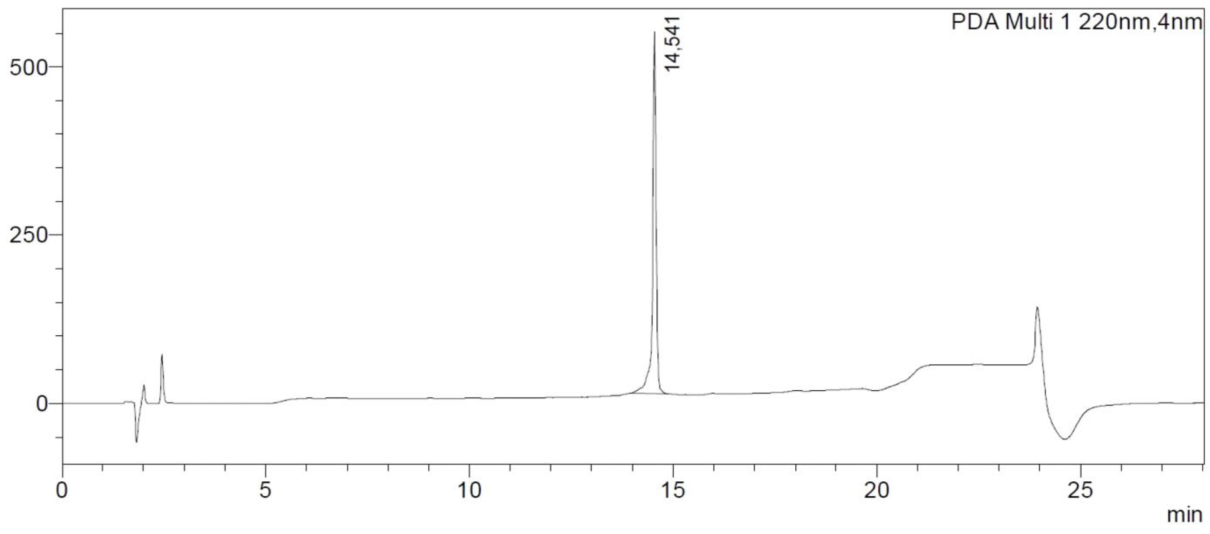

Figure S10. Analytical HPLC chromatogram of **11**, recorded using Shimadzu Nexera Liquid Chromatograph (SPD-M20A/DGU-20A3R/LC-20AD) with a Phenomenex Kinetex C18 column (150 × 4.6 mm, 5 µm, 100 Å). UV detection was performed at 220 nm. Retention time (t_R): 14.5 min [elution: linear gradient 10-70% MeCN (0.1% TFA) in H_2O (0.1% TFA) over 15 min; flow rate: 1 mL/min].

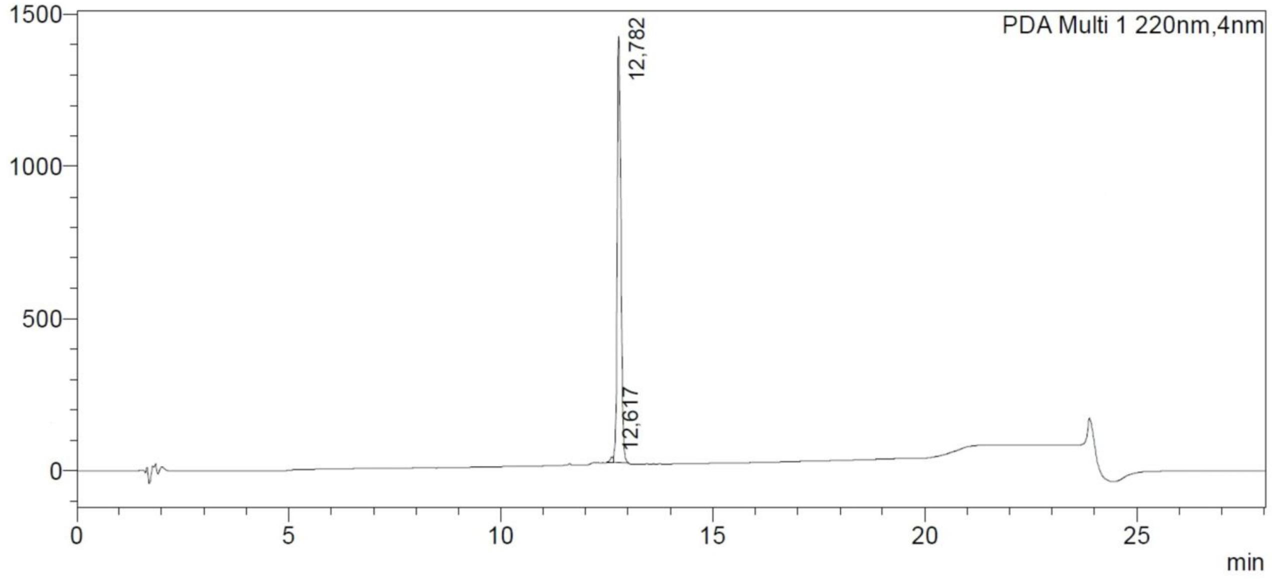

PDA Ch1 220nm

Peak#	Ret. Time	Area	Height	Area%
1	12,617	88180	17111	1,035
2	12,782	8434898	1402104	98,965
Total		8523078	1419216	100,000

Figure S11. Analytical HPLC chromatogram of **12**, recorded using Shimadzu Nexera Liquid Chromatograph (SPD-M20A/DGU-20A3R/LC-20AD) with a Phenomenex Kinetex C18 column (150 × 4.6 mm, 5 µm, 100 Å). UV detection was performed at 220 nm. Retention time (t_R): 12.8 min [elution: linear gradient 10-70% MeCN (0.1% TFA) in H_2O (0.1% TFA) over 15 min; flow rate: 1 mL/min]. Purity >98%.

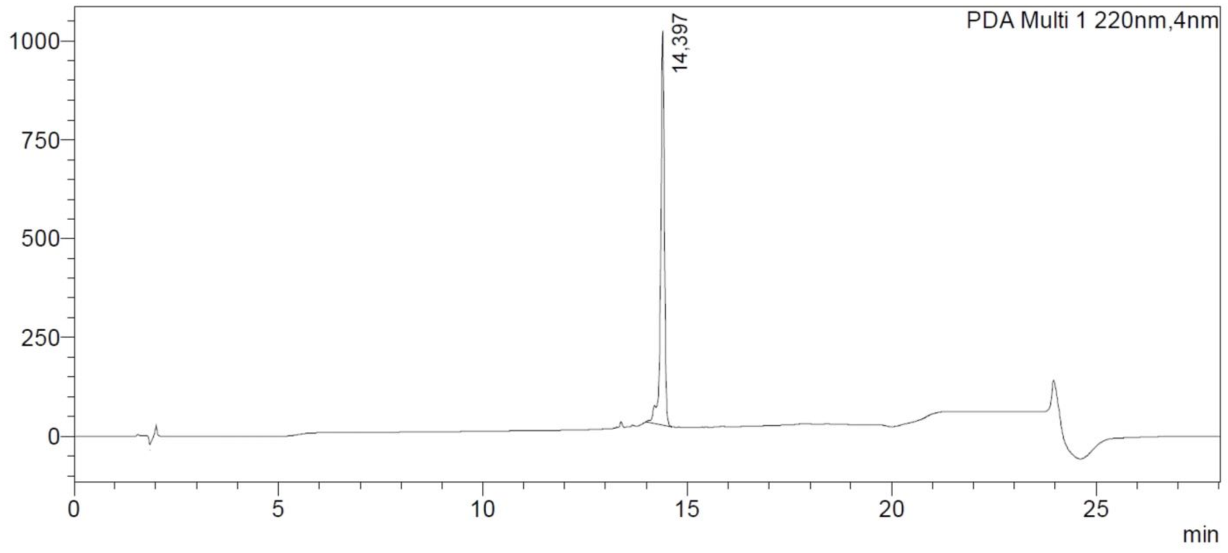

Figure S12. Analytical HPLC chromatogram of **13**, recorded using Shimadzu Nexera Liquid Chromatograph (SPD-M20A/DGU-20A3R/LC-20AD) with a Phenomenex Kinetex C18 column (150 × 4.6 mm, 5 µm, 100 Å). UV detection was performed at 220 nm. Retention time (t_R): 14.4 min [elution: linear gradient 10-70% MeCN (0.1% TFA) in H_2O (0.1% TFA) over 15 min; flow rate: 1 mL/min].

*MS spectra of guanidino-based cyclic TL-derived peptides (**2-13**).*

(A)

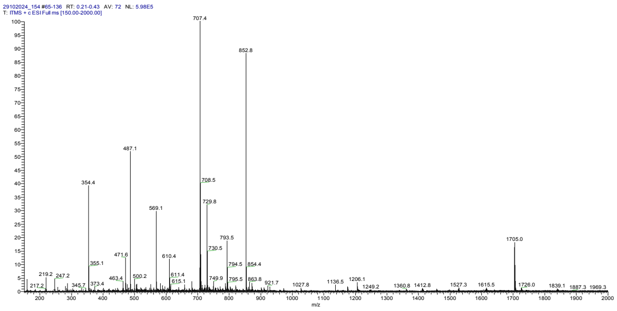

(B)

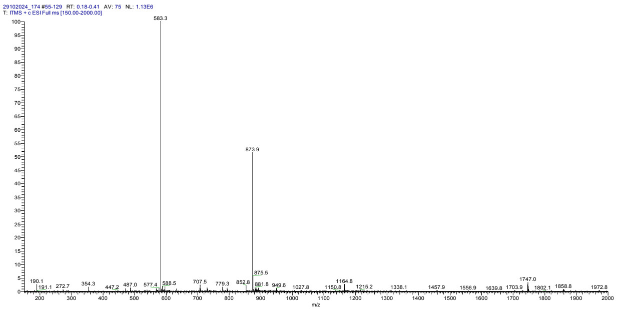

(C)

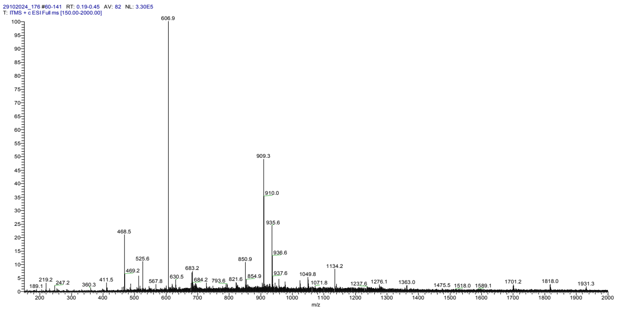

(D)

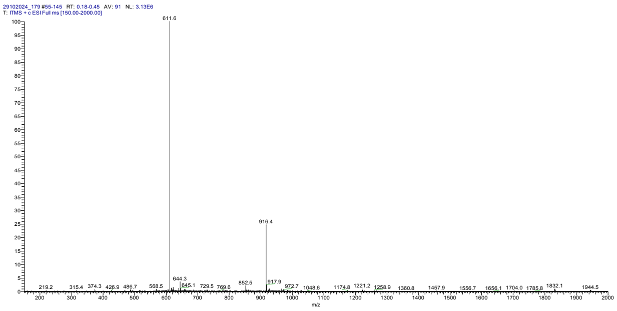

(E)

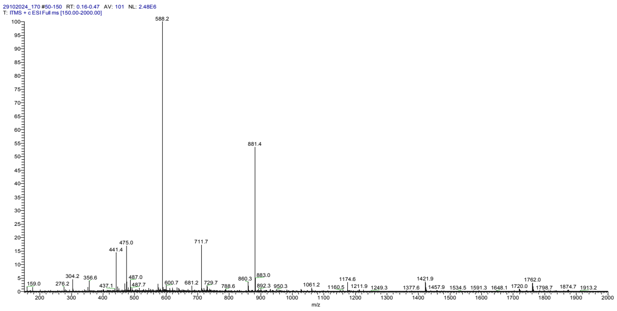

(F)

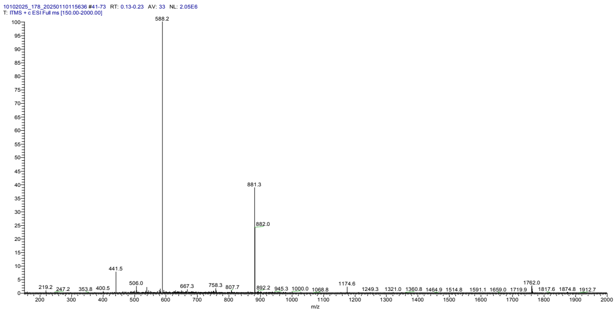

(G)

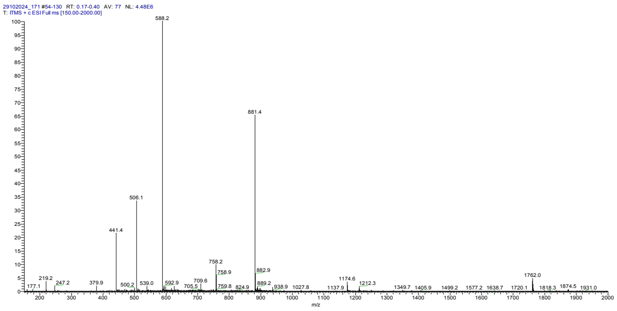

(H)

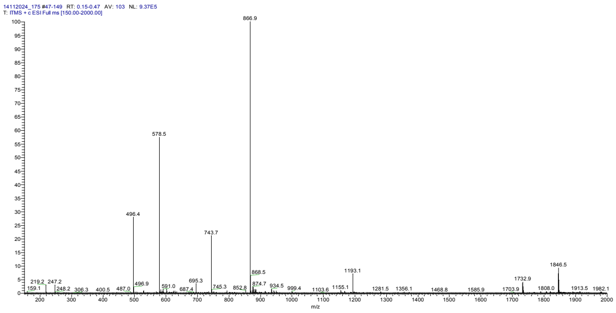

(I)

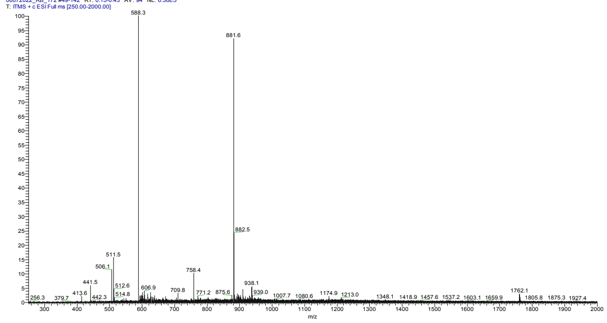

(J)

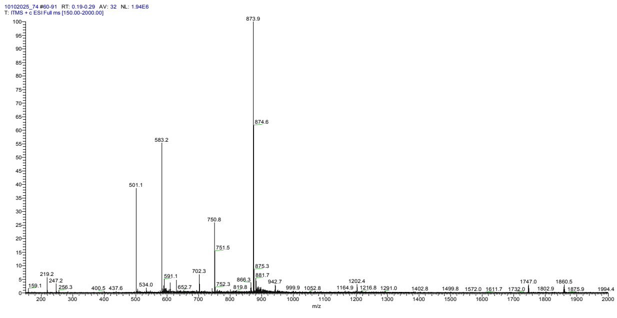

(K)

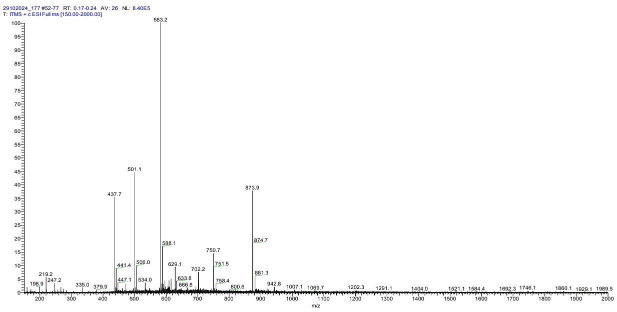

(L)

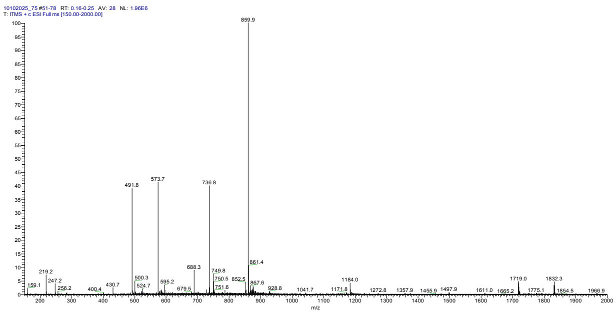

Figure S13. MS spectra for **2** (A), **3** (B), **4** (C), **5** (D), **6** (E), **7** (F), **8** (G), **9** (H), **10** (I), **11** (J), **12** (K), **13** (L).

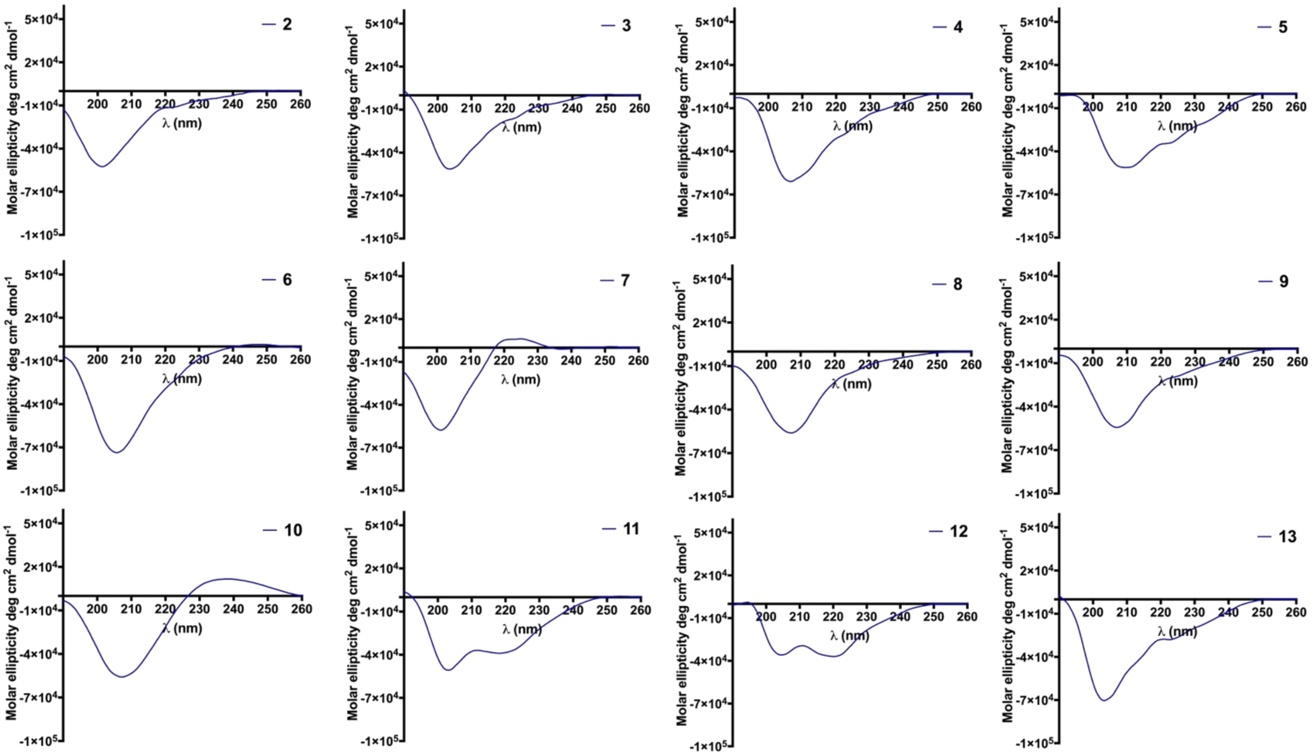

Figure S14. CD spectra of guanidino-based cyclic TL-derived peptides (**2-13**).

Table S2. Cytotoxicity against HaCaT cells after 2 h exposure.

	Cell viability (%)[a]					
**Peptide**	**3.12**	**6.25**	**12.5**	**25**	**50**	**100**
**2**	102 (±2)	96 (±3)	95 (±3)	95 (±2)	64 (±3)	7 (±1)
**3**	126 (±12)	137 (±2)	125 (±4)	104 (±4)	52 (±1)	5 (±1)
**4**	100 (±1)	100 (±1)	100 (±1)	47 (±1)	7 (±2)	8 (±2)
**5**	128 (±3)	126 (±3)	128 (±5)	76 (±4)	18 (±1)	4 (±1)
**6**	102 (±2)	101 (±1)	101 (±2)	101 (±2)	81 (±4)	13 (±1)
**7**	101 (±2)	100 (±1)	102 (±1)	72 (±3)	16 (±2)	8 (±3)
**8**	111 (±5)	112 (±1)	115 (±1)	110 (±1)	92 (±1)	63 (±4)
**9**	106 (±1)	106 (±1)	98 (±3)	94 (±2)	74 (±1)	22 (±5)
**10**	105 (±1)	110 (±2)	112 (±3)	112 (±2)	101 (±2)	82 (±2)
**11**	98 (±2)	98 (±3)	86 (±2)	57 (±2)	35 (±2)	10 (±1)
**12**	101 (±1)	103 (±2)	88 (±2)	47 (±1)	11 (±2)	10 (±2)
**13**	107 (±2)	104 (±1)	76 (±1)	31 (±1)	7 (±1)	2 (±1)

[a]Cytotoxicity assays performed at 2 h revealed a generally similar trend to that observed after 24 h, with all peptides maintaining high cell viability (>80%) up to 12.5 µM. Moderate decreases were detected at 25 µM for some analogues (notably **4**, **11-13**), whereas peptides **2**, **6-8** and **10** remained largely well tolerated even at 50 µM. The overall profile confirms a consistent and time-dependent cytotoxic behavior, with prolonged exposure (24 h) accentuating peptide-induced effects but without significant changes in relative toxicity ranking among the analogues.

Table S3. Cytotoxicity of peptides **2**, **6**, **7** and **12** against BEAS-2B cells after 2 and 24 h exposure.

Peptide	**Cell viability** (%)[a]											
	**3.12**		**6.25**		**12.5**		**25**		**50**		**100**	
	2 h	*24 h*	*2 h*	*24 h*	*2 h*	*24 h*	*2 h*	*24 h*	*2 h*	*24 h*	*2 h*	*24 h*
**2**	**100** (±8)	**132** (±9)	**107** (±1)	**119** (±9)	**100** (±9)	**114** (±8)	**91** (±8)	**102** (±11)	**26** (±4)	**49** (±6)	**5** (±2)	**8** (±2)
**6**	**111** (±2)	**104** (±2)	**119** (±2)	**111** (±1)	**114** (±3)	**111** (±2)	**100** (±2)	**107** (±7)	**30** (±2)	**33** (±6)	**2** (±1)	**4** (±1)
**7**	**105** (±2)	**111** (±2)	**117** (±1)	**104** (±3)	**80** (±1)	**90** (±6)	**28** (±2)	**34** (±2)	**6** (±2)	**9** (±5)	**2** (±1)	**4** (±3)
**12**	**106** (±5)	**93** (±2)	**104** (±3)	**86** (±2)	**56** (±4)	**66** (±9)	**14** (±3)	**14** (±4)	**4** (±1)	**2** (±1)	**4** (±1)	**0** (±0)

Table S4. Membrane fluidity evaluation calculating the GP value on LUVs mimicking Gram-positive and Gram-negative membranes.

	$GP_{laurdan}$		
**Cmpd**	**DOPG/CL**	**DOPG/CL + cmpd[a]**	**DOPG/CL+cmpd[b]**
**2**	-0.16±0.11	-0.11±0.05	0.01±0.01
**6**	-0.16±0.11	-0.10 ±0.03	0.03±0.02
**7**	-0.16±0.11	-0.07±0.01	0.04±0.03
**12**	-0.16±0.11	-0.04±0.05	0.04±0.01

	$GP_{laurdan}$		
**Cmpd**	**DOPG/DOPE/CL**	**DOPG/DOPE/CL + cmpd[a]**	**DOPG/DOPE/CL + cmpd[b]**
**2**	-0.21±0.10	-0.18±0.03	0.07±0.01
**6**	-0.21±0.10	-0.05±0.05	0.05±0.02
**7**	-0.21±0.10	-0.01±0.02	0.04±0.02
**12**	-0.21±0.10	-0.06±0.05	0.06±0.01

cmpd[a]:concentration is 25μM. cmpd[b]: concentration is 50 μM.

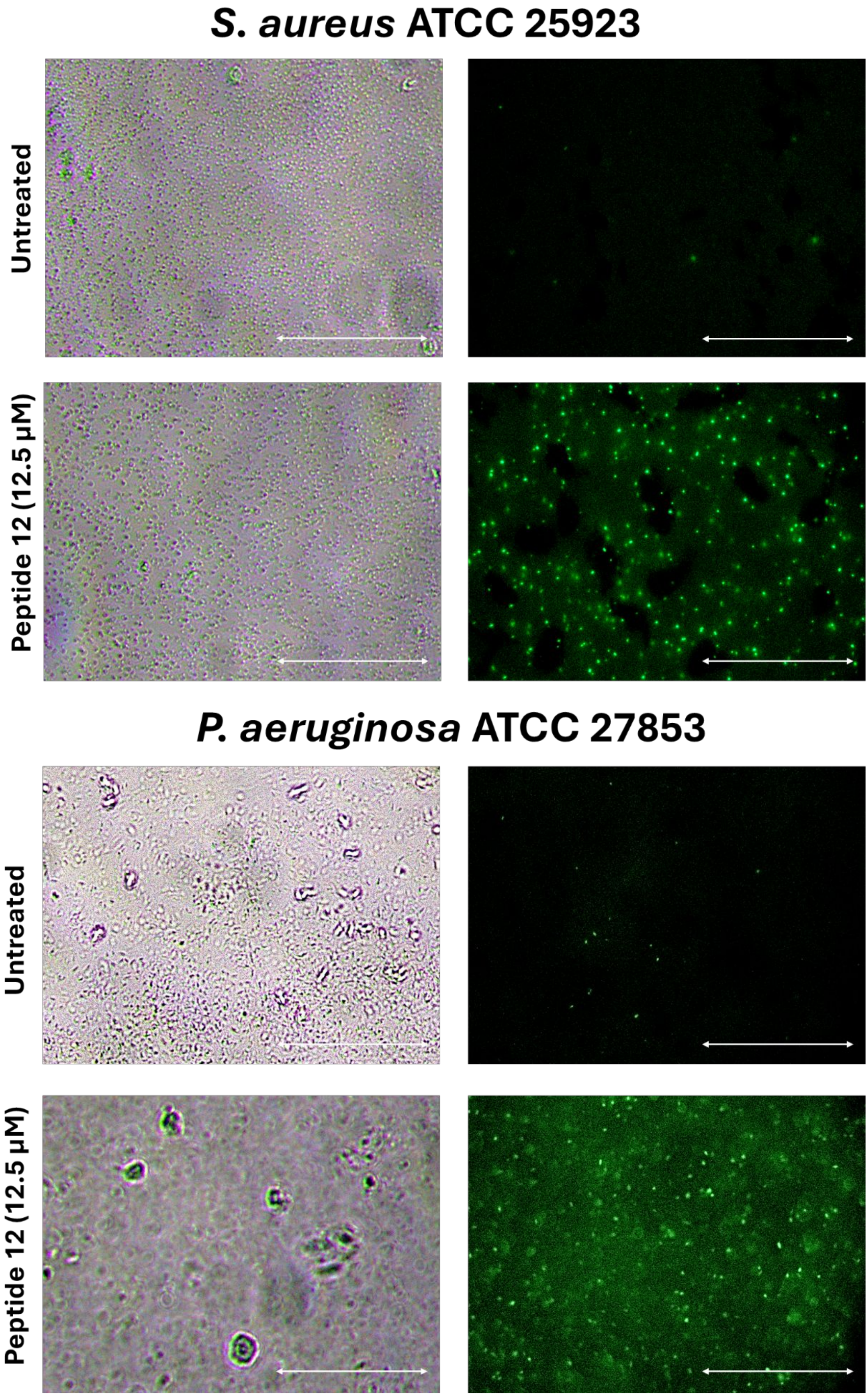

Figure S15. Phase-contrast (left) and fluorescence images (right) showing the effect of peptide **12** on membrane permeability of *S. aureus* ATCC 25923 and *P. aeruginosa* ATCC 27853 to SYTOX Green. SYTOX Green (1 µM) was added prior peptide addition. Peptide **12** was tested at 12.5 µM for 10 minutes. Scale bar = 100 µm.
